# Contingent intramuscular boosting of P2XR7 axis improves motor function in transgenic ALS mice

**DOI:** 10.1007/s00018-021-04070-8

**Published:** 2021-12-22

**Authors:** Paola Fabbrizio, Jessica D’Agostino, Cassandra Margotta, Giulia Mella, Nicolò Panini, Laura Pasetto, Eliana Sammali, Flavia Raggi, Gianni Sorarù, Valentina Bonetto, Caterina Bendotti, Giovanni Nardo

**Affiliations:** 1 Laboratory of Molecular Neurobiology, Department of Neuroscience, Istituto di Ricerche Farmacologiche Mario Negri IRCCS, Via Mario Negri 2, 20156 Milan, Italy; 2 Laboratory of Antitumor Pharmacology, Department of Oncology, Istituto di Ricerche Farmacologiche Mario Negri IRCCS, Via Mario Negri 2, 20156 Milan, Italy; 3 Laboratory of Translational Biomarkers, Department of Biochemistry and Molecular Pharmacology, Istituto di Ricerche Farmacologiche Mario Negri IRCCS, Via Mario Negri 2, 20156 Milan, Italy; 4 Department of Neuroscience, Azienda Ospedaliera di Padova, Via Giustiniani 2, 35128 Padua, Italy

**Keywords:** Amyotrophic lateral sclerosis, Mouse models, Skeletal muscle, Satellite cells, Macrophages, Myogenesis

## Abstract

Amyotrophic lateral sclerosis is a fatal neurodegenerative disorder that leads to progressive degeneration of motor neurons and severe muscle atrophy without effective treatment. Most research on the disease has been focused on studying motor neurons and supporting cells of the central nervous system. Strikingly, the recent observations have suggested that morpho-functional alterations in skeletal muscle precede motor neuron degeneration, bolstering the interest in studying muscle tissue as a potential target for the delivery of therapies. We previously showed that the systemic administration of the P2XR7 agonist, 2′(3′)-*O*‐(4-benzoylbenzoyl) adenosine 5-triphosphate (BzATP), enhanced the metabolism and promoted the myogenesis of new fibres in the skeletal muscles of SOD1G93A mice. Here we further corroborated this evidence showing that intramuscular administration of BzATP improved the motor performance of ALS mice by enhancing satellite cells and the muscle pro-regenerative activity of infiltrating macrophages. The preservation of the skeletal muscle retrogradely propagated along with the motor unit, suggesting that backward signalling from the muscle could impinge on motor neuron death. In addition to providing the basis for a suitable adjunct multisystem therapeutic approach in ALS, these data point out that the muscle should be at the centre of ALS research as a target tissue to address novel therapies in combination with those oriented to the CNS.

**Supplementary Information:**

The online version contains supplementary material available at 10.1007/s00018-021-04070-8.

## Introduction

Amyotrophic lateral sclerosis (ALS) is a fatal motor neuron (MN) disease characterised by degenerative changes in upper and lower motor neurons [1, 2]. Symptoms typically occur in late middle life and present as relentlessly progressive muscle atrophy and weakness, with the effects on respiratory muscles limiting survival to 2–4 years after disease onset in most cases [1, 3]. ALS is the most common adult MN disease with an incidence of 2 per 100,000 and a prevalence of 5.4 per 100,000 individuals [4]. The current treatment options are based on the symptom management and respiratory support with the only approved medications in widespread use, Riluzole and Edaravone, providing only modest benefits and only in some patients [1].

While most ALS cases are sporadic (sALS), about 10% are familial (fALS) and characterised by autosomal dominant inheritance [5]. The genetic causes of fALS, with approximately 40–55% of cases, accounted for variants in known ALS-linked genes [6]. Although more than 50 potentially causative or disease-modifying genes have been identified, pathogenic modifications in SOD1, TARDBP, FUS and C9ORF72 occur most frequently, with disease-causing variants in other genes being relatively uncommon [5]. However, the diagnostic advancements have only helped explain a fraction of sALS cases, with the aetiology remaining unexplained in over 90% of patients [7].

Over the last 20 years, the use of mutant SOD1 (mSOD1) mice has allowed identifying several mechanisms that contribute to MN injury [8]. It has emerged that certain aspects of ALS are noncell-autonomous and that other cell types within the spinal cord, including microglia, astrocytes and T cells, contribute to the progression of the disease [9–11]. However, this remarkable body of knowledge did not yield the expected outcomes in terms of therapeutic benefits suggesting that MN protection alone is insufficient to prevent peripheral axons and muscles from degenerating [12–14].

The denervation atrophy of skeletal muscles is an early event in ALS, which anticipates MN death of several weeks [15–18]. Studies in transgenic mSOD1 mice showed that muscle atrophy occurs as early as 50 days postnatal when MNs do not show signs of degeneration [15]. Besides, the restricted induction of mSOD1 in skeletal muscles causes denervation, atrophy and MN loss, indicating that skeletal muscle is likely to concur to the pathogenesis in ALS [19, 20]. This evidence has led to ALS being reviewed as a distal axonopathy, whereby skeletal muscle contributes to a retrograde signalling cascade that degrades MNs [21–23].

Several preclinical and clinical studies support the antagonism of the purinergic P2X receptor 7 (P2XR7), a family member of purinergic ionotropic receptors, in ALS pathogenesis [24]. However, studies on transgenic ALS mice produced controversial results probably related to the multifaceted activity of P2XR7 [25]. Indeed, P2XR7 induction results in a plethora of downstream effects, including releasing pro-inflammatory mediators or activating cell proliferation/differentiation pathways, which are transposable based on the tissue context and the time of P2XR7 activation [26–30]. Therefore, the systemic pharmacological inhibition of the P2XR7 was ineffective in counteracting the disease progression or slightly improved the survival of SOD1G93A mice [24, 31, 32].

Conversely, SOD1G93A mice lacking the P2XR7 showed a remarkable worsening of motor ability with an anticipated clinical onset hinting an essential role of P2XR7 in the peripheral system during the disease pathoprogression [33]. Indeed, P2XR7 activation mediates Schwann cell proliferation and remyelination following nerve injury [34, 35] and promotes myogenesis in the skeletal muscles [36]. In keeping with this, we recently found that the activation of P2XR7 by its agonist, 2′(3′)-*O*‐(4-benzoylbenzoyl) adenosine 5-triphosphate (BzATP) within the skeletal muscles of SOD1G93A mice enhanced the metabolism of myofibres and myogenesis, thus ameliorating the denervation atrophy of hindlimb skeletal muscles [37].

In this work, we further investigated the effects of P2XR7 activation in SOD1G93A mice producing novel and definitive evidence for a protective role of the purinergic signalling in the muscular tissue. Namely, the intramuscular administration of BzATP improved the motor performance of ALS mice through a direct and immune-mediated preservation of the skeletal muscle that retrogradely propagated to CNS.

## Materials and methods

### Reagents

Unless otherwise stated, BzATP and all other reagents were from Sigma Aldrich. A-804598 was purchased from Tocris Bioscience. Interferon-gamma (INFy) and interleukin-4 (IL-4) were obtained from PeproTech and Mouse-M-CSF from MACS Miltenyi Biotec.

### Animals

Female and male transgenic SOD1G93A mice on C57BL/6JOlaHsd genetic background (C57-SOD1G93A) and corresponding nontransgenic (Ntg) littermates were used in this study. 12 weeks-old C57-SOD1G93A mice were randomly grouped and subjected to intramuscular injection of vehicle (PBS) or P2XR7 most potent agonist BzATP, at the dose of 1 or 10 mg/kg/muscle, twice a week. The animals were sacrificed two hours after the last i.m. injection at 13 weeks (presymptomatic stage, no recordable motor impairment; No. 5 for each group), 18 weeks (onset of motor impairment as assessed by the Paw Grip strength test; No. 4 BzATP-treated mice, No. 5 vehicle-treated mice) or 21 weeks (symptomatic stage; when non-treated mice show ~ 60% reduction in motor activity as assessed by the Paw Grip strength test; No. 12 BzATP-treated mice, No. 10 vehicle-treated mice). Disease progression was monitored bi-weekly, starting from ten weeks of age, in BzATP-treated and vehicle SOD1G93A transgenic mice. Bodyweight and paw grip strength were recorded for each session, as previously described [38, 39].

The longitudinal bodyweight loss was calculated as the ratio between the bodyweight of a single mouse at the different time points and its maximum weight achieved.

The Paw Grip strength test involved placing the mouse on the wire-lid of a conventional housing cage. The mice are placed on a horizontal grid at 30 cm from the table and the tail is gently pulled until they grasp the grid with their fore and hind paws. The lid is gently turned upside down and the latency time of the mouse to fall on the table is recorded for a maximum of 90 s. Each mouse is given up to three attempts and the most prolonged latency is recorded. The onset of hind limb force motor deficit is considered when the mice showed the first signs of impairment (latency less than 90 s) in the Paw Grip strength test.

For the evaluation of the P2XR7 muscular levels, female C57-SOD1G93A mice, female 129SvHsd-mSOD1 (129 Sv-SOD1G93A) mice, female B6;SJL-Tg(Thy1-TARDBP)4Singh/J (Jackson; Stock No: 012836), female Tg(Prnp-FUS)WT3Cshw/J (Jackson; Stock No: 017916) mice and relative Ntg mice were sacrificed to the presymptomatic (No. 4 C57-SOD1G93A: 13 weeks; No. 3 129 Sv-SOD1G93A: 12 weeks; No. 5 Thy1-TARDBP: 1.14 weeks; No. 3 PrP-hFUS: 3 weeks), onset (No. 4 C57-SOD1G93A: 18 weeks; No. 4 129 Sv-SOD1G93A: 14 weeks; No. 4 Thy1-TARDBP: 2 weeks; No. 3 PrP-hFUS: 4 weeks) and symptomatic (No. 3 C57-SOD1G93A: 21 weeks; No.4 129 Sv-SOD1G93A: 16 weeks; No. 4 Thy1-TARDBP: 3 weeks; No. 3 PrP-hFUS: 6 weeks) disease stages.

The IRFMN adheres to the principles set out in the following laws, regulations and policies governing the care and use of laboratory animals: Italian Governing Law (D.lgs 26/2014; Authorisation n.19/2008-A issued March 6, 2008 by Ministry of Health); Mario Negri Institutional Regulations and Policies providing internal authorisation for persons conducting animal experiments (Quality Management System Certificate—UNI EN ISO 9001:2015—Reg. N° 6121); the NIH Guide for the Care and Use of Laboratory Animals (2011 edition) and EU directives and guidelines (EEC Council Directive 2010/63/UE). In addition, the ethical procedure has been approved by the Animal Welfare Office, Department of Public Health and Veterinary, Nutrition and Food Safety, General Management of Animal Care and Veterinary Drugs of the Italian Ministry of Health (protocol number 79/2020PR).

All efforts were made to minimise animal suffering and use the minimum number of animals necessary to obtain reliable results. The animals were housed under specific pathogen-free (SPF) standard conditions (22 ± 1 °C, 55 ± 10% relative humidity and 12-h light/dark schedule), 3–4 per cage, with free access to food (standard pellet, Altromin, MT, Rieper) and water.

### Human skeletal muscle biopsies

All skeletal muscle biopsies were selected from the Biobank of the Neuromuscular Bank of Tissues at the University of Padua (Telethon Network of Genetic Biobanks; TNGB). Samples were frozen into the liquid phase of the isopentane, previously cooled in liquid nitrogen, for no more than 45 s. Frozen muscles were then stored at − 80 °C until use. The monthly ALSFRS-R slope [progression rate to last visit (PRL) = 48-ALSFRS-R score at last visit/ disease duration from the onset to the last visit] was used to define the rate of disease progression of ALS patients [40].

### SDS PAGE and immunoblot assay

Equal amounts of total protein lysates were obtained by homogenisation of tissue muscles, sciatic nerves (SN) and human samples in homogenisation buffer as previously described [38, 39]. Cell cultures were harvested in SDS Laemmli sample buffer. To analyse protein components, samples were run on Mini-PROTEAN^®^ TGX™ Gels (BioRad) and transferred onto PVDF membranes (BioRad). Following the saturation with blocking agent, blots were incubated overnight at 4 °C with the specified antibody against anti-P2XR7, rabbit (1:500; Alomone); anti-Pax7, mouse (1:1000; DSHB); anti-MyoG, mouse (1:350; DSHB); anti-Myod, rabbit (1:1000; Proteintech), anti-p44/42 MAPK (ERK1/2) (L34F12) mouse antibody (1:1000; Cell Signaling Technology Inc.) and anti-phospho-p44/42 MAPK (ERK1/2) (Thr202/Tyr204), rabbit (1:1000; Cell Signaling Technology Inc.), anti-pAKT, rabbit (1:1000; Cell Signaling Technology Inc.), anti-AKT, rabbit (1:1000; Cell Signaling Technology Inc.) anti- pGSK, rabbit (1:1000; Cell Signaling Technology Inc.) and anti-GSK, rabbit (1:2000; Cell Signaling Technology Inc.); anti-GFAP, mouse (1:10000 Chemicon); anti-ARG rabbit (1:500; Abcam) anti-mannose receptor, rabbit (1:500; Abcam) and anti-NF200, rabbit (1:4000; Abcam); anti-MBP, rat (1:1000; BioRad). Primary antibodies were then detected with HRP-conjugated secondary antibodies and visualised through the Chemi-Doc XRS System (BioRad) using Luminata Forte Western Chemiluminescent HRP Substrate (Millipore). Protein levels were normalised to the total amount of protein detected by the Stain-Free membrane activation system (BioRad) or GAPDH using anti-GAPDH, mouse (1:10000; Sigma-Aldrich).

### Real-time PCR

Total RNA from TA, GCM and QC muscles was extracted in Trizol (Ambion), purified (PureLink RNA mini kit, Invitrogen) and quantified with the spectrophotometer (NanoDrop 1000 Spectrophotometer V3.7).

The Taq Man (Applied Biosystems) gene expression assay was used following the manufacturer’s instructions on triplicate cDNA samples, using the 1X Universal PCR (Life Technologies) master mix and the 1X mix containing specific receptor probes. The following probes were used: nicotinic cholinergic receptor, gamma subunit (AChRγ) (CHRNG; Mm00437419_m1; Life Technologies); insulin growth factor 1 (Igf1; Mm00439560_m1; Life Technologies); interleukin 10 (Il-10; Mm00439614_m1; Life Technologies); Tumor necrosis factor-alpha (TNF-alpha—Mm00443258_m1; Life Technologies). The relative quantification was calculated from the ratio between the number of cycles (Ct) at which the signal exceeded a threshold set within the logarithmic phase of the given gene and that of the reference actin gene (4310881E; Life Technologies). The mean values of the tripled results for each animal were used as individual data for the 2^−ΔΔCt^ statistical analysis.

### Primary satellite cell cultures

Satellite cell isolation and labelling were performed as described in Mozzetta et al. [41]. Briefly, hindlimb muscles were isolated from sacrificed mice and digested for 45 min at 37 °C under agitation in phosphate-buffered saline (PBS) (Sigma-Aldrich) supplemented with Dispase II (2.4 U/ml; Roche), Collagenase A (2 mg/ml; Roche), 0.4 mM CaCl2, 5 mM MgCl2 and deoxyribonuclease I (DNase I) (10 μg/ml; Roche). Cell suspensions were resuspended in HBSS and filtered with 100-μm and 40-μm filters. Single-cell suspension was stained with CD45/CD31/Ter119 phycoerythrin (PE) for lineage exclusion, Sca1 (Stem cell antigen 1)-fluorescein isothiocyanate (FITC) and α7 integrin allophycocyanin (APC). Cells were sorted using Moflo Astrios (Beckman Coulter).

SCs were seeded on Matrigel-coated plates (Corning) at low density (3500 cells/cm^2^) and cultured in Cyto-Grow (Resnova) complete medium as a growth medium (GM) for four days. For myogenic differentiation, after reaching the confluence, SCs have been shifted in DMEM + 2% horse serum up to 48 h, in the presence or absence of drug treatment.

### Primary macrophage cultures

Primary MФ cultures were obtained as previously described [42]. Briefly, single-cell suspension was obtained following spleen harvest by mechanical tissue dissociation in RCB buffer (NH4Cl 150 mM, NaHCO3 10 mM and EDTA 1 mM). Cells were plated (4 × 10^6^/ml) in RPMI, 10% fetal bovine serum, 100 U/ml gentamycin, 100 µg/ml streptomycin and 100 U/ml penicillin. After 2 h, nonadherent cells were removed and medium enriched with 10 ng/ml mouse macrophage colony-stimulating factor (Sigma-Aldrich). After one week, cells were used for western blotting and immunofluorescence analysis. For M1 differentiation, MФ were incubated 24 h with 20 ng/ml IFNγ (PeproTech) and 1ug/ml LPS from Escherichia coli 055:B5 (Sigma Aldrich). For M2 polarisation, cells were stimulated with IL-4 (PeproTech) 10 ng/ml.

### Immunofluorescence assays

Ex vivo tissues: mice were perfused with 0.1 M PBS. Tissues were quickly dissected out. QC and SN were snap-frozen in liquid nitrogen whilst the SC was left in 4%PFA overnight at 4 °C, rinsed and stored 24 h in 30% sucrose and 0.01 M PBS.

Muscles: 20 μm longitudinal and 10 μm transversal serial QC cryosections were collected on poly-lysine objective slides (VWR International). To evaluate the cross-sectional area (CSA), serial transverse QC sections were fixed in cold acetone solution for 10’ and stained with Wheat Germ Agglutinin, Alexa Fluor™ 488 Conjugate (1:500; Thermo Fisher) and Hoechst (1:1000; Roche). Longitudinal muscle QC sections were fixed in acetone for 10’, air-dried and washed. After blocking with 10% NGS or NDS in PBS for 1 h, muscle slides were incubated overnight with primary antibodies: anti-CD11b, mouse (1:200; BioRad); anti-iNOS, rabbit (1:200; Invitrogen); anti-mannose receptor, rabbit (1:500; Abcam) and anti-P2XR7, goat (1:200; MyBioSource) at 4 °C. Secondary antibodies were as follows with Alexa488 anti-rabbit, Alexa564 anti-goat and Alexa647 anti-mouse (1:500; Thermo Fisher). The nuclei were counterstained whit Hoechst (1:1000; Roche).

Spinal Cord and SN were cut in 30 µm and 20 µm serial transverse sections, respectively. The following primary antibodies and staining were used: anti-GFAP, mouse (1:2500; Chemicon); anti-Iba1, rabbit (1:200; Waco); anti-MBP, rat (1:1000; BioRad). Anti-NF200, rabbit (1:4000; Abcam); anti-ChAT, goat (1:200; Sigma-Aldrich). Secondary antibodies were as follows with Alexa488 anti-mouse, Alexa488 anti-rat, Alexa488 anti-goat, Alexa594 anti-rabbit, Alexa647 anti-rabbit and Alexa647 anti-mouse (1:500; Thermo Fisher).

In vitro: cells were fixed with 4% PFA in PBS for 15’ and permeabilised with 0.1% Triton for 5’. Unspecific signals were blocked with 1% BSA for 30’. Primary antibodies anti-MF20, mouse (1:50; DSHB), anti-CD11b, rat (1:200; BioRad); anti-mannose receptor, rabbit (1:500; Abcam) and anti-Ki67, rabbit (1:200; Abcam). All Abs were diluted in blocking solution and incubated overnight at 4 °C. Cells were incubated with secondary antibodies, Alexa488 anti-rabbit, Alexa488 anti-mouse and Alexa647 anti-rat (1:500; Thermo Fisher), for 1 h at room temperature and washed in PBS. Nuclei were counterstained whit Hoechst (1:1000; Roche) in PBS and glasses were mounted in Fluorsave Mountant (Calbiochem).

Images were acquired with an Olympus virtual slide system VS110 (Olympus, Center Valley, USA) at 20X magnification and analysed through ImageJ (U.S. National Institute of Health) or using a sequential scanning mode by an A1 Nikon confocal running NIS Elements at 20X or 40X magnification.

### Image analysis

MN survival: Fluorescence-labelled sections images (No. 10 per animal) of the Spinal Cord were analysed with an A1 Nikon confocal running NIS Elements (Nikon) and acquired at 20X magnification. For the MN count analysis, a total of twelve serial ChAT-stained sections were analysed with Nikon confocal running NIS Elements at 20X magnification. The neuron areas were analysed with ImageJ (U. S. National Institutes of Health). Only neuronal somas with an area ≥ 400 μm^2^ were considered for quantitative analysis of MN numbers.

Astrogliosis: The same approach was used to evaluate the astrocytosis and microgliosis staining by calculating the percentage of covered area (Area fraction %) per field for each section analysed with Fiji software. Muscle CSA was determined using MuscleJ [43].

Tissue MФ analysis: a stereological random sampling procedure was applied as previously described [38]. Briefly, a grid of rectangular sampling fields was delineated on the profile of the muscle slice using the "Grid" function in ImageJ (U. S. National Institutes of Health). To ensure that each part of the tissue slice had the same probability of being sampled, the analysis was done on defined Z-stacked image fields, acquired with A1 Nikon confocal running NIS Elements (Nikon) at 20X magnification, at a fixed distance between them.

In vitro: the same approach described above was used to evaluate the proliferation and differentiation of SCs. SC proliferation was evaluated for each well on stereological image fields acquired with A1 Nikon confocal running NIS Elements (Nikon) at 40X magnification by counting the number of DAPI + /Ki67 + (Anti-Ki67; Abcam) cells per field. SC differentiation will be assessed by evaluating the fusion index given by the number of nuclei within myotubes stained with anti-MyHC (MF20- myocytes containing ≥ two nuclei/total MF20 + cells). The percentage of differentiated cells was calculated as follows: (nuclei within MF20- myocytes/total number of nuclei). Image fields were acquired with A1 Nikon confocal running NIS Elements (Nikon) at 20X magnification.

### Ex vivo *cell proliferation assay*

Twelve weeks-old SOD1G93A mice were treated with BzATP or PBS, i.m. for two times/week following the protocol described below (three animals for each group). The animals were sacrificed and the hindlimb muscles were dissected to isolate the SCs as described above.

SCs were seeded onto 24-well plates at a density of 7000 cells in a standard growth medium (Cyto-Grow; Resnova). Each well was acquired at 10X magnification in a bright field and the index of proliferation was evaluated after four days by counting the cell number with ImageJ (US National Institutes of Health).

### Statistics

GraphPad Prism v.9.01 (GraphPad Software) was used for the statistical analysis. For each analysis, the dependent and group variable are respectively named on the *y*- and *x*-axis of the graph.

The sample size for behavioural analysis was defined according to the "Guidelines for preclinical animal research in ALS/MND: A consensus meeting" [44]. The Mantel–Cox log-rank test and Kaplan–Meier survival plots were used for comparing the onset of motor dysfunction (phenotype) between groups. The mice were collected through a block-randomisation in which the blocks are defined on the body weight (Supplementary Table 1), sex and sibling separation to avoid confounding adjustments. The dependent variable was the age (in weeks) at the euthanasia, and the independent variable was the treatment. Paw grip strength and body weight were analysed by repeated-measures ANOVA with Sidak’s post-analysis checking for normality in the residual and homoscedasticity through the Geisser–Greenhouse’s epsilon to evaluate potential violations. Parametric unpaired *t* test and the one-way ANOVA with Tukey’s post-analysis were used to compare the differences between two or more mouse groups, respectively. D’Agostino and Pearson omnibus normality test and relative QQ plots were used to assess the assumption of normality. All experiments were done a minimum of three times. *P* < 0.05 was considered statistically significant. Further details, including *P* values and number of samples, are documented in the Results, Figures and relative captions.

## Results

### Intramuscular administration of BzATP ameliorates the disease progression of SOD1G93A mice

In vivo intraperitoneal treatment in SOD1G93A mice with the P2XR7 specific agonist, BzATP benefited the skeletal muscles. From a clinical point of view, the systemic administration of BzATP could lead to the development of undesirable side effects associated with the pro-inflammatory activity of the P2XR7 signalling. In this study, we directly administered BzATP in the hindlimbs skeletal muscles of SOD1G93A mice to bypass this issue. *Tibialis* *Anterior* (TA), *Gastrocnemius* *Medialis* (GCM) and *Quadriceps* (QC) were treated with a BzATP dose proportional to the local expression of the P2XR7 receptor registered in transgenic mice at 12wks of age (Supplementary Fig. 1a, b). Accordingly, 1 mg/kg/muscle BzATP, dissolved in 10 μl of 0.1 M PBS, was injected in TA and GCM muscles, which showed on average, respectively, a fourfold and sixfold induction of P2XR7 as compared to Ntg littermates, whilst 10 mg/kg BzATP, dissolved in 10 μl of 0.1 M PBS, was administered to the QC muscles that exhibit no significant P2XR7 upregulation.

**Fig. 1:**
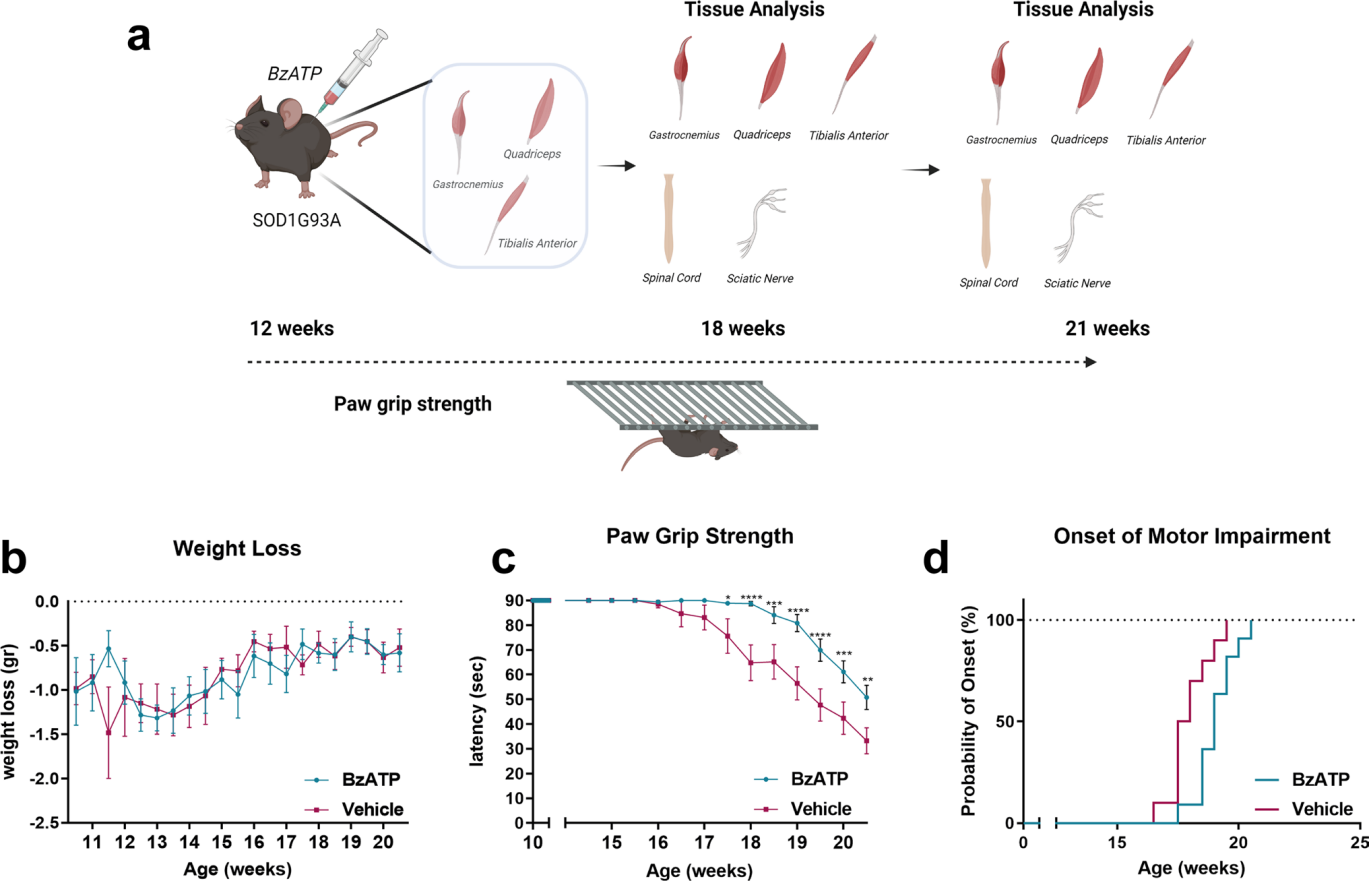
BzATP intramuscular administration delayed the disease course of SOD1G93A mice. **a** Schematic representation of the experimental design of the study. *Image* *Created* *in* *Biorender.com*. **b** Body weight loss and **c** Paw Grip Endurance (PaGE) test for BzATP- (*n* = 12) and PBS- (*n* = 10) treated SOD1G93A mice. The data are reported as mean ± SEM for each time point. *P < 0.05, ****P < 0.0001 by repeated-measures ANOVA with Sidak’s post-analysis. **d** BzATP-treated mice had a delayed onset of motor impairment than PBS-treated mice. *P* < 0.0129 by Mantel-Cox log-rank test

Following this protocol, SOD1G93A mice were treated with BzATP or PBS twice a week starting from 12 weeks of age (Fig. 1a). This schedule was selected as muscle atrophy is a very early event in SOD1G93A mice and is already overt at the time of treatment. Four mice per group were sacrificed at the disease onset (18 weeks). Twelve and ten mice were treated, respectively, with BzATP or PBS and monitored until the symptomatic disease stage (21 weeks) (Fig. 1a).

During the treatment, no difference in weight loss was observed between the two experimental groups, excluding any significant side effect upon the induction of P2XR7 in ALS mice (Fig. 1b). Notably, in the BzATP treated mice, muscle strength impairment was delayed and progressed slower up to 20 weeks of age than the control group (Fig. 1c): this was translated in the postponement of the onset of motor impairment of ~ one week compared with the control group (Vehicle = 17.95 ± 0.86 weeks, BzATP = 19.05 ± 0.82 weeks) (Fig. 1d).

### The P2XR7 boosting in the hind limb skeletal muscles of SOD1G93A mice delayed the denervation atrophy

The impairment of skeletal muscles is an early event in the pathogenic cascade [15–18], pivotal in determining the motor disability of SOD1G93A mice. We previously found that in vivo intraperitoneal administration of BzATP prevented the denervation atrophy of skeletal muscles in SOD1G93A mice.

In keeping with the ameliorated clinical phenotype, we found that the hind limb skeletal muscles of BzATP-treated mice were less compromised than controls during the disease progression.

**Fig. 2:**
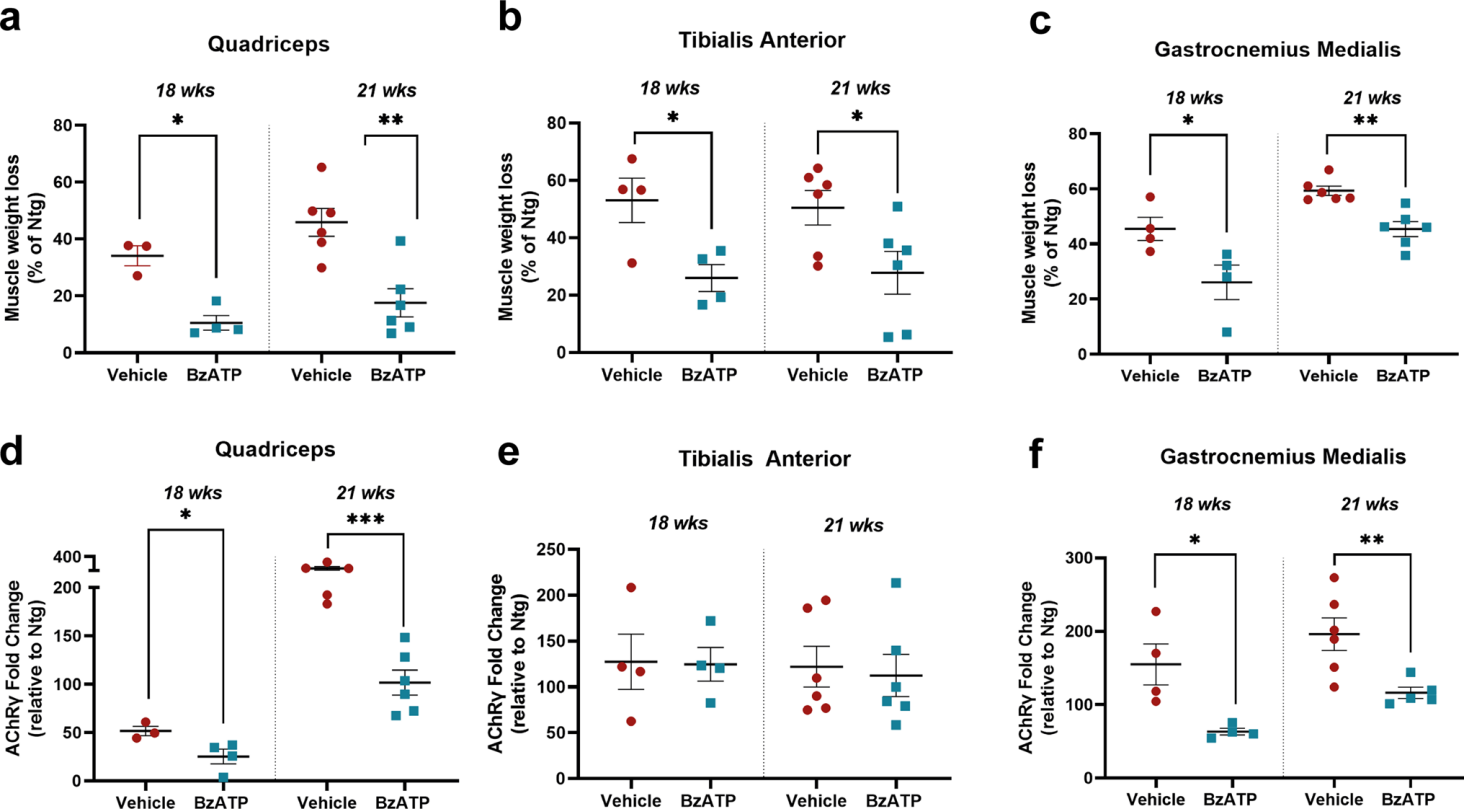
BzATP intramuscular administration reduced the denervation atrophy of hind limb skeletal muscles of SOD1G93A mice. **a**–**c** Muscle wasting was calculated at 18 and 21 weeks of age by measuring of the **a** Quadriceps (QC), **b** Tibialis anterior (TA) and **c** Gastrocnemius Medialis (GCM) muscle weight of BzATP- and PBS-treated SOD1G93A mice compared to Ntg littermates. The data are presented as mean ± SEM. The independent experiments are scattered on the graph at each time-point, for each experimental group. *P < 0.05, **P < 0.01, ***P < 0.001 by unpaired *t* test. **d**–**f** Real-time PCR for AChR-γ transcript in the **d** QC, **e** TA and **f** QC muscle of BzATP- and PBS-treated SOD1G93A mice and the corresponding Ntg littermates at 18 and 21 weeks of age. The data are normalised to β-actin and expressed as the mean ± SEM fold change ratio between BzATP- and PBS-treated SOD1G93A mice compared to Ntg littermates. The independent experiments are scattered on the graph at each time-point, for each experimental group. *P < 0.05, **P < 0.01, ***P < 0.001 by unpaired *t* test

At 18 weeks, we recorded a reduction in the muscle mass of the QC, TA and GCM, respectively, of 34.1 ± 6.0%, 53.1 ± 15.4% and 45.48 ± 8.4% compared to Ntg littermates, which decreased at 10.53 ± 5.1%, 26.03 ± 9.4% and 26.13 ± 12.5% upon the BzATP treatment (Fig. 2a–c).

Notably, muscle mass preservation extended at 21 weeks of age with PBS-treated SOD1G93A mice showing a reduction in the muscle mass of the QC, TA and GCM, respectively, of 45.87 ± 12%, 50.48 ± 14.7% and, 59.33 ± 4.2% compared with non-transgenic (Ntg) littermates, which decreased at 17.57 ± 12%, 27.82 ± 18.3% and 45.40 ± 6.6% in the BzATP-treated SOD1G93A mice (Fig. 2a–c). Moreover, a significant decrease in the levels of the foetal γ-subunit of the acetylcholine receptor (AChRγ) mRNA was recorded upon BzATP treatment in the QC (Fig. 2d) and GCM (Fig. 2f) but not TA muscles (Fig. 2e) at both 18 and 21 weeks of age, indicating longitudinal preservation of hind limb muscle innervation compared with PBS-treated mice [45].

### The P2XR7 boosting in the hind limb skeletal muscles of SOD1G93A mice elicited satellite cell proliferation and differentiation

We previously showed that BzATP enhanced the activation of myogenic satellite cells in skeletal muscles of mSOD1 mice [37]. Therefore, we next assessed the impact of the BzATP administration on the rate of myofibres differentiation in the QC of the two experimental mouse groups during the disease progression. The QC was selected based on a higher percentage of preservation than TA and GCM in BzATP-treated mice as compared to PBS-treated mice.

**Fig. 3:**
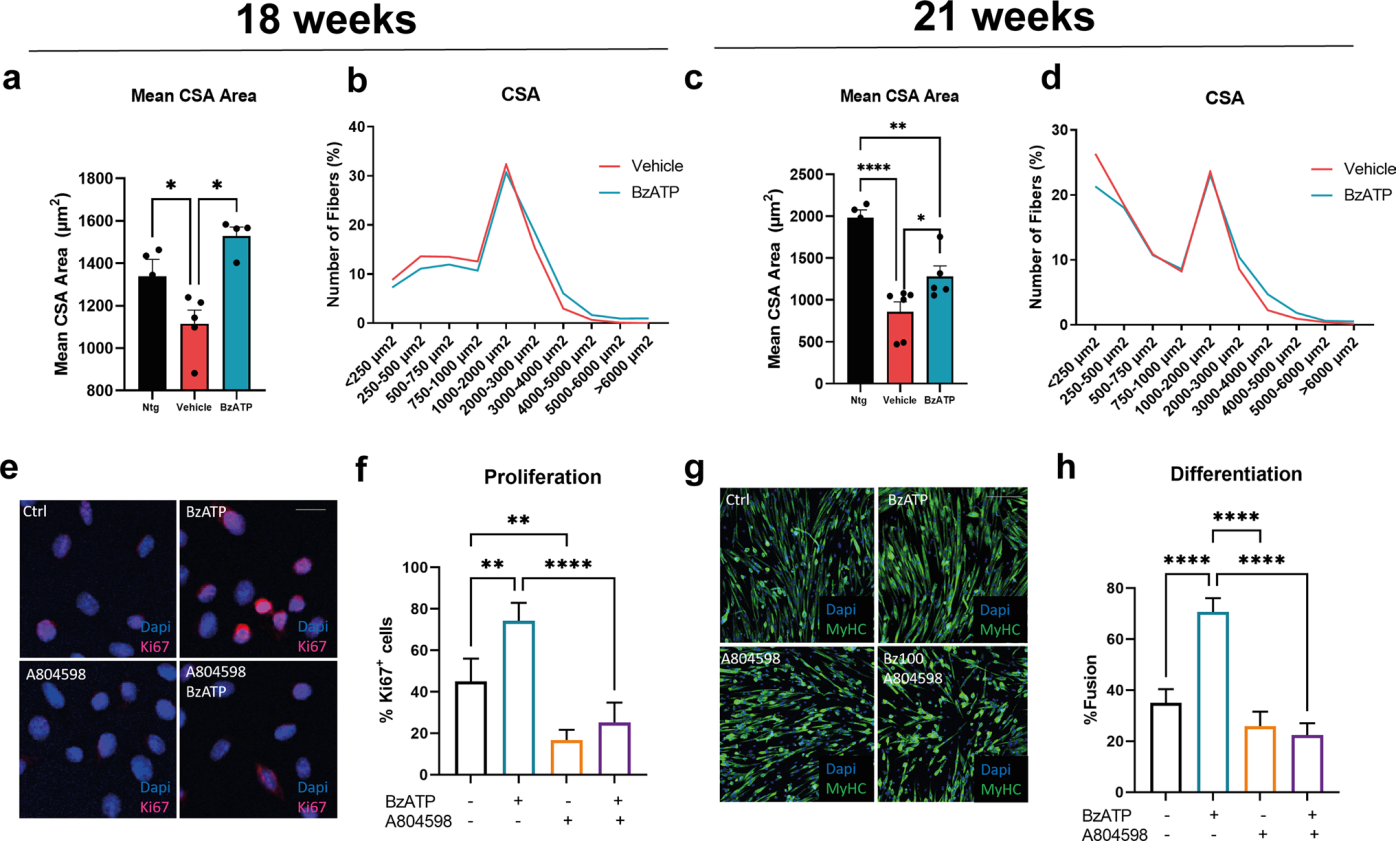
BzATP intramuscular administration improves the rate of muscle fibre differentiation by enhancing the activity of satellite cells. **a** Mean cross-sectional area (CSA) and **b** frequency distribution of muscle fibres in BzATP-treated SOD1G93A mice compared to vehicle mice at 18 and 21 weeks of age. BzATP increases the dimension of fibres, which is associated to a shift towards muscle fibres with a large size. The data are presented as mean ± SEM of *n* = 4 and *n* = 5 independent experiments for each group at 18 and 21 weeks of age, respectively. *P < 0.05, **P < 0.01, ****P < 0.001 by one-way ANOVA with Tukey’s post-analysis. **e** Representative confocal images showing the immunostaining for Ki67 (red) and DAPI (blue) on primary SOD1G93A SC cultures exposed to 100 µM BzATP and/or 10 µM A804598 for 24 h in GM. Scale bar = 50 µm. **f** The proliferation index was assessed by counting the number of Ki67 and DAPI juxtaposition in primary SOD1G93A SC cultures simultaneously. **g** Representative confocal images showing the immunostaining for MF20-MyHC (green) and DAPI (blue) on primary SOD1G93A SC cultures treated with 100 µM BzATP and/or 10 µM A804598 in DM for 48 h. Scale bar = 100 µm. **h** The fusion index was calculated as (No. nuclei present in MyHC + cells with two or more nuclei/No. myotubes. In f and h, the data are reported by means ± SEM from three independent experiments for each group. **P < 0.01, ***P < 0.001, ****P < 0.0001 by one-way ANOVA with Tukey’s post-analysis

We found that BzATP-treated mice had a greater muscle fibre cross-sectional area than controls at both the disease onset (Fig. 3a) and symptomatic stage (Fig. 3c) as an effect of an increased percentage of fibres with a large-size compared to fibres with a small size (Fig. 3b, d).

We next assessed the impact of the BzATP boosting on the expression of two critical myogenic factors in the QC muscle of 18 weeks-old C57SOD1G93A mice: Pax7, the hallmark of satellite cell (SC) stemness [46], and Myogenin (MyoG), a marker of early commitment and differentiation [47]. MyoG, but not Pax7, was more upregulated in the QC muscle of BzATP-treated mice than Vehicle-treated mice (Supplementary Fig. 2a–c). Moreover, our analysis revealed that the Myoblast Determination protein 1 (MyoD), a transcription factor critical at defining the activated SCs fate [48], was significantly upregulated upon P2XR7 boosting (Supplementary Fig. 2a, d).

Given the myogenic proliferative effect in the skeletal muscle of transgenic mice upon BzATP administration, we next assessed the effect of BzATP on primary SCs isolated from SOD1G93A mice.

We first evaluated the proliferative index of ex vivo isolated SCs upon intramuscular BzATP administration to 12 weeks-old SOD1G93A for one week, following the protocol illustrated above.

SCs were isolated and plated to an established density (38.4 cell/mm^2^) after the treatment. On the fourth day in the growth medium, SCs from animals pre-treated with BzATP increased the proliferation index of + 1.9% compared to SCs isolated from PBS-treated animals (489.6 ± 61.43 and 257.2 ± 26.48 cells/mm^2^) (Supplementary Fig. 3a, b). Then, we evaluated the protein levels of myogenic proliferation markers by immunoblot. In agreement with the higher cell proliferation, we found an increase in Pax7 (Supplementary Fig. 3c, d) and MyoD (Supplementary Fig. 3c, e) in BzATP pre-treated SCs compared to vehicle, and this correlated with a heightened in the P2XR7 (Supplementary Fig. 3c, f).

Based on the evidence, we next evaluated, in vitro*,* the direct pharmacological effect of BzATP on primary SOD1G93A SCs.

The immunohistochemistry against the nuclear protein Ki67 revealed a higher proliferative rate of SCs following the treatment with 100 μM BzATP. This effect was abolished following the pre-treatment of SCs with the P2XR7 antagonist A804598 (Fig. 3e, f). We next set up a differentiation assay on ex vivo cultures of SOD1G93A SCs to evaluate the capability of BzATP in promoting their differentiation. Following 100 μM BzATP treatment, SCs showed a higher differentiation index as assessed by the higher percentage of myocytes with two or more nuclei than controls. This effect was reverted upon P2XR7 antagonism (Fig. 3g, h). Previous studies on nucleotide receptors revealed that P2XR7 activates signalling cascades characteristic of trophic factors [49, 50]. Among them, the inhibition of the glycogen synthase kinase 3 (GSK3), the P2XR7-related calcium influx coupled to the extracellular signal-regulated kinase 1/2 (ERK½) or the canonical phosphatidylinositol 3-kinases **(**PI3K)/protein kinase B (PKB, aka AKT) signalling can directly promote cell survival and proliferation [49].

Because of this information, we next investigated whether one of these pro-survival/regenerative pathways could be elicited in the skeletal muscle of SOD1G93A mice following BzATP administration. Given the activation speed of these axes, we assessed the degree of GSK3, AKT and ERK½ phosphorylation in the QC of 12 weeks-old female SOD1G93A mice one week after the administration of 10 mg/Kg BzATP (two times/week).

**Fig. 4:**
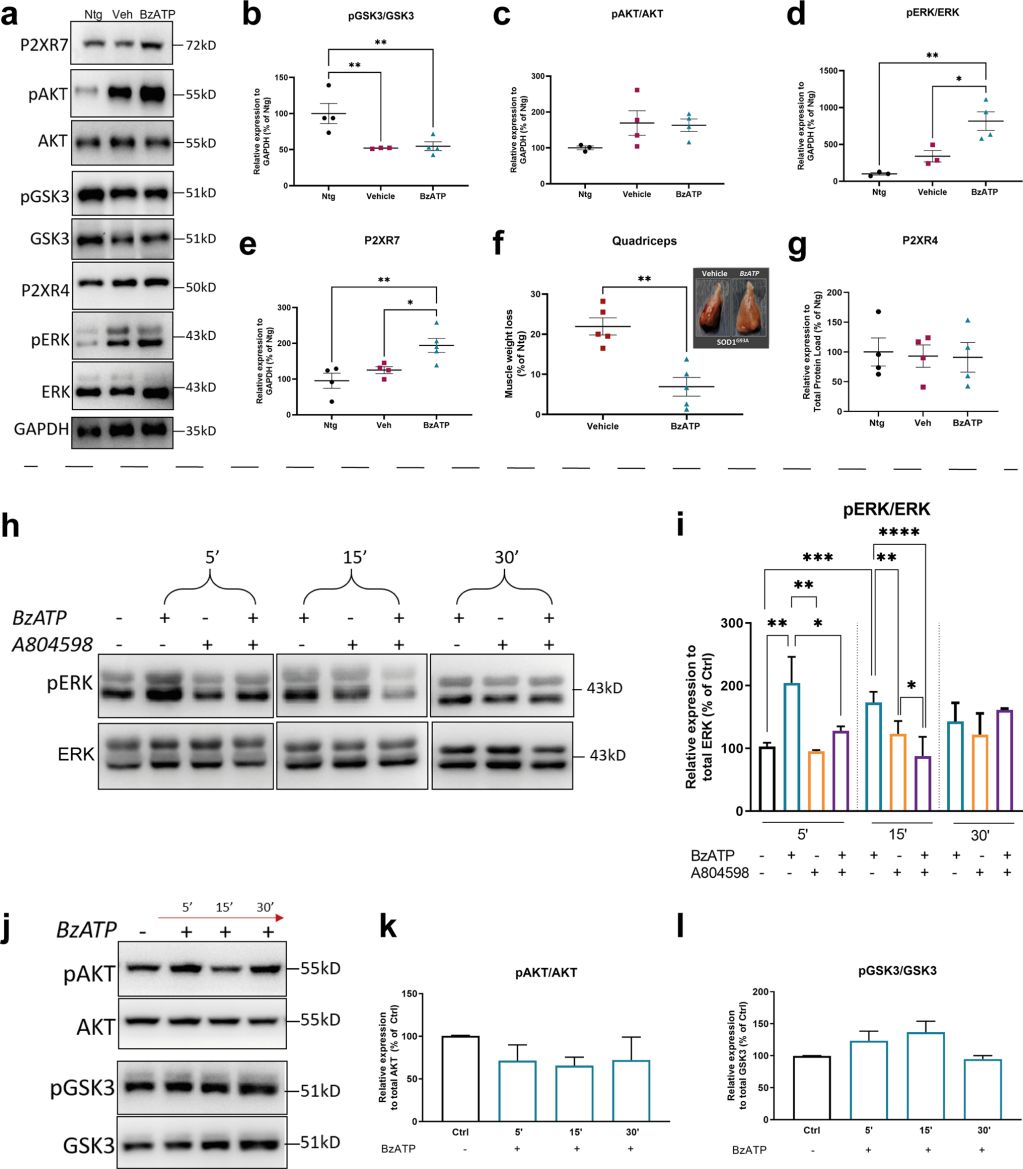
BzATP administration reduce muscle atrophy by eliciting the MAPK ERK1/2 signalling in the skeletal muscle of SOD1G93A mice. **a**–**e** Representative Immunoblot images and densitometric analysis of **a**, **b** pGSK3/GSK3, **a**, **c** pAKT/AKT, **a**, **d** pERK/ERK, **a**, **e** P2XR7 and **a**, **g** P2XR4 expression in QC extracts from BzATP- and PBS-treated SOD1G93A mice compared to Ntg littermates at 12 weeks of age. The data are reported as percentages of the relative Ntg (mean ± SEM). *P < 0.05, **P < 0.01 by one-way ANOVA with Tukey’s post-analysis. **f** Representative images of the QC muscles of BzATP- and PBS-treated SOD1G93A mice at 12 weeks of age showing increased muscle size in BzATP-treated SOD1G93A mice, which have a lower muscle wasting. The data are reported as percentages of the relative Ntg (mean ± SEM). The independent experiments for each experimental group are scattered on the graph. **P < 0.01 by one-way ANOVA with unpaired *t* test. **h**, **i** Representative Immunoblot images and densitometric analysis of pERK/ERK in primary SOD1G93A SCs exposed to 100 μM BzATP for 5 min, 15 min or 30 min with or without 10 μM A804598. **j**–**l** Representative Immunoblot images and densitometric analysis of **j**, **k** pAKT/AKT and **j**, **l** pGSK3/GSK3 in primary SOD1G93A SCs exposed to 100 μM BzATP for 5’, 15’ or 30’. The data are reported as percentage of the relative Ntg (mean ± SEM) of three independent experiments for each group. **P < 0.01, ***P < 0.001, ****P < 0.0001 by one-way ANOVA with Tukey’s post-analysis

We found reduced activation of GSK3 in both treated mice and vehicles compared to Ntg littermates (Fig. 4a, b), whilst no substantial variation was associated with AKT (Fig. 4a, c). On the contrary, ERK½ phosphorylation significantly heightened in the QC of BzATP-treated mice compared to PBS-treated mice (Fig. 4a, d), and this was associated with increased P2XR7 protein levels (Fig. 4a, e) and a lower QC muscle wasting than PBS-treated SOD1G93A (BzATP-treated: 6.9 ± 5.1%; Vehicles: 21.94 ± 4.7%) (Fig. 4f). Noteworthy, the same effect was registered also in 12 weeks-old, BzATP-treated SOD1G93A male mice (Supplementary Fig. 4).

To evaluate whether the activity of BzATP was actually mediated by P2XR7, we investigated the expression levels of P2X4R. Unlike P2XR7, P2X4R showed no significant variation between BzATP-treated and PBS-treated mice suggesting that the BzATP dosage used was specific for P2XR7 (Fig. 4g).

To further confirm this evidence, we evaluated the rate of ERK½ phosphorylation in ex vivo isolated primary SC culture derived from SOD1G93A mice exposed to 100 μM BzATP for 5’, 15’ and 30’. We found higher P2XR7 levels on the cell membrane of BzATP-treated than untreated SCs (Supplementary Fig. 5) and a time-dependent activation of ERK½ with a peak at 5’ minutes. This effect was inhibited by the pre-treatment of SC culture with the P2XR7 antagonist A804598 (Fig. 4h, i). No difference in the phosphorylation level of GSK3 and AKT was observed upon BzATP administration (Fig. 4j–l).

### Boosting the P2XR7 signalling promotes the polarisation of infiltrating macrophages to M2 fingerprint in the hind limb skeletal muscles of SOD1G93A mice

The P2XR7 receptor is expressed by different immune cells, including macrophages (MФ) [51]. MФ are the most abundant immune cells recruited following muscle injury, as they are pivotal to promote muscle regeneration [52]. M1-MФ infiltrate early to promote the clearance of necrotic debris and the activation of SCs, whereas M2-MФ appear later to sustain tissue healing through the differentiation of new myofibres [52, 53].

It was previously reported that purinergic signalling regulates both M1 and M2 macrophage function at different levels by controlling the secretion of cytokines, phagocytosis and the production of reactive oxygen species [54].

**Fig. 5:**
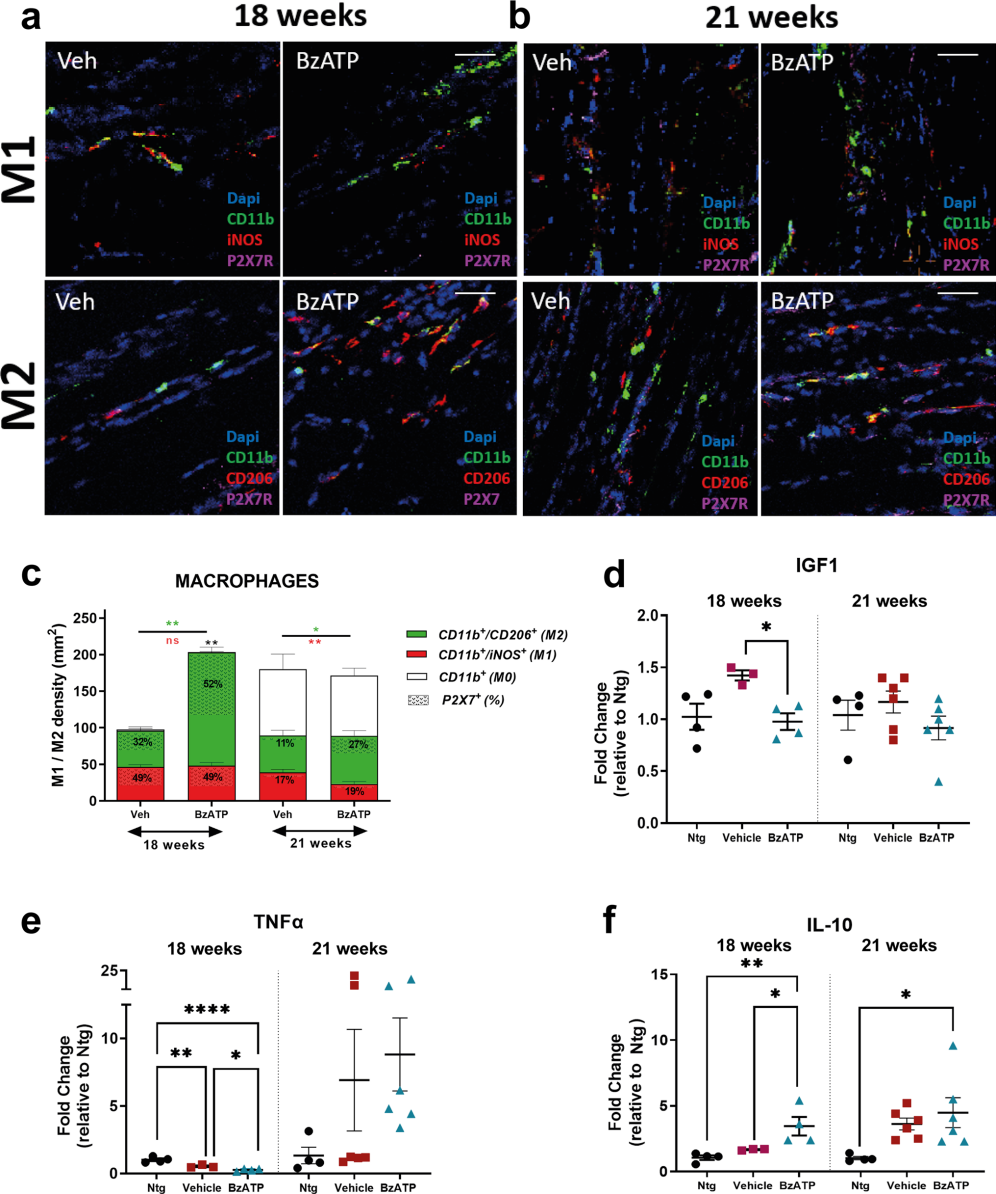
BzATP intramuscular administration in SOD1G93A mice influences the rate of macrophage infiltration and their polarisation towards and M2-like phenotype in the skeletal muscle. **a**, **b** Representative confocal images of longitudinal section of QC muscle of BzATP- and PBS-treated SOD1G93A mice at **a** 18 and **b** 21 weeks of age showing the distribution of M1-MΦ (CD11b + , iNOS +) and M2-MΦ (CD11b + , CD206 +) expressing the P2XR7. Scale bar = 100 µm. **c** Quantification of MΦ infiltration and fingerprint within the QC of BzATP- and PBS-treated SOD1G93A mice at **a** 18 and **b** 21 weeks of age. The shaded area on the histograms are the percentage of M1-MΦ or M2-MΦ expressing the P2XR7. The data are reported as (mean ± SEM) from at least four independent experiments for each group. **P < 0.01, **P < 0.01, by unpaired *t* test. **d**–**f** Real-time PCR for **d** Igf1, **e** Tnfα and **f** Il-10 transcript in the QC muscle of BzATP- and PBS-treated SOD1G93A mice at 18 weeks and 21 weeks. The data are normalised to β-actin and expressed as the mean ± SEM fold change ratio between BzATP- and PBS-treated SOD1G93A mice compared to Ntg littermates. The independent experiments for each experimental group are scattered on the graph at each time point.*P < 0.05, **P < 0.01, ****P < 0.0001 by one-way ANOVA with Tukey’s post-analysis

Based on this information, we next investigated the effect of the activation of the P2XR7 signalling on MФ recruitment and polarisation in the skeletal muscle of SOD1G93A mice. We found an increased density of CD11b^+^ cells within the QC muscle of BzATP-treated mice compared with the control group. This effect was significant at the disease onset but not at 21 weeks of age, when the MФ had already massively infiltrated the skeletal muscle of mSOD1 mice (Fig. 5a–c).

We next investigated the inflammatory fingerprint acquired by MФ within the skeletal muscle of SOD1G93A mice upon BzATP induction. The histological analysis at 18 weeks of age revealed no difference among the experimental groups in the density of the M1 iNOS^+^ myeloid cells infiltrated in the QC muscle upon P2XR7 boosting with BzATP. In contrast, a remarkable increase of the M2 CD206^+^ counterpart was recorded compared with the controls (Fig. 5a, c). Notably, 52% of MФ with an M2-biased phenotype expressed P2XR7 in BzATP-treated mice versus 32% in control mice. Conversely, the 49% of MФ with an M1-biased phenotype expressed P2XR7 in both the BzATP- and PBS-treated animals (Fig. 5a, c). Albeit with a smaller entity than the onset, an increased density of M2- CD206^+^ was still detectable at 21 weeks of age in the face of a reduced number of M1-iNOS^+^ pro-inflammatory MФ in the QC muscle of BzATP-treated mice compared to PBS-treated mice (Fig. 5b, c). At this stage, the 27% of MФ with an M2-biased phenotype expressed P2XR7 in BzATP-treated mice versus the 11% in vehicle mice. Conversely, the 19% and the 17% of MФ with an M1-biased phenotype expressed P2XR7, respectively, in the BzATP- and PBS-treated animals (Fig. 5b, c).

The analysis of inflammatory milieu in the QC muscle of BzATP-treated mice compared with the vehicle group at the disease onset revealed a significant downregulation of *Insulin-like* *Growth* *Factor* *1* (*Igf1*) (Fig. 5d), a cytokine released by M1-macrophages exerting an autocrine function pivotal to trigger the M2 gene program [55] and of *Tumor* *Necrosis* *Factor* *α* (*Tnfα*) (Fig. 5e). In keeping with this, BzATP-treated mice compared to vehicles showed an increase in the *Interleukin* *10* (*IL-10*) (Fig. 5f), whose overexpression in skeletal muscle is essential to promote the MФ polarisation shift toward the anti-inflammatory phenotype and SC differentiation to myocyte [53, 56].

At the advanced disease stage, no variation was registered in the inflammatory response between the experimental groups surmising exhaustion of the immune response and the relative effects in the hind paw muscles of mSOD1 mice (Fig. 5d–f).

Because extracellular ATP accumulates at sites of tissue injury and serves as a danger molecule, expression of P2XR7 by MФ suggests that P2XR7 engagement may govern MФ functions. To test this possibility, we examined whether blocking the P2XR7 using A804598, a receptor-specific antagonist [24], would inhibit macrophage polarisation to M2 cells. We cultured spleen–derived SOD1G93A MФ under either M1- or M2-polarizing conditions. In those cultures, A804598 was added to block P2XR7 and induction of M2 associated molecules was determined 24 h later by immunoblot.

**Fig. 6:**
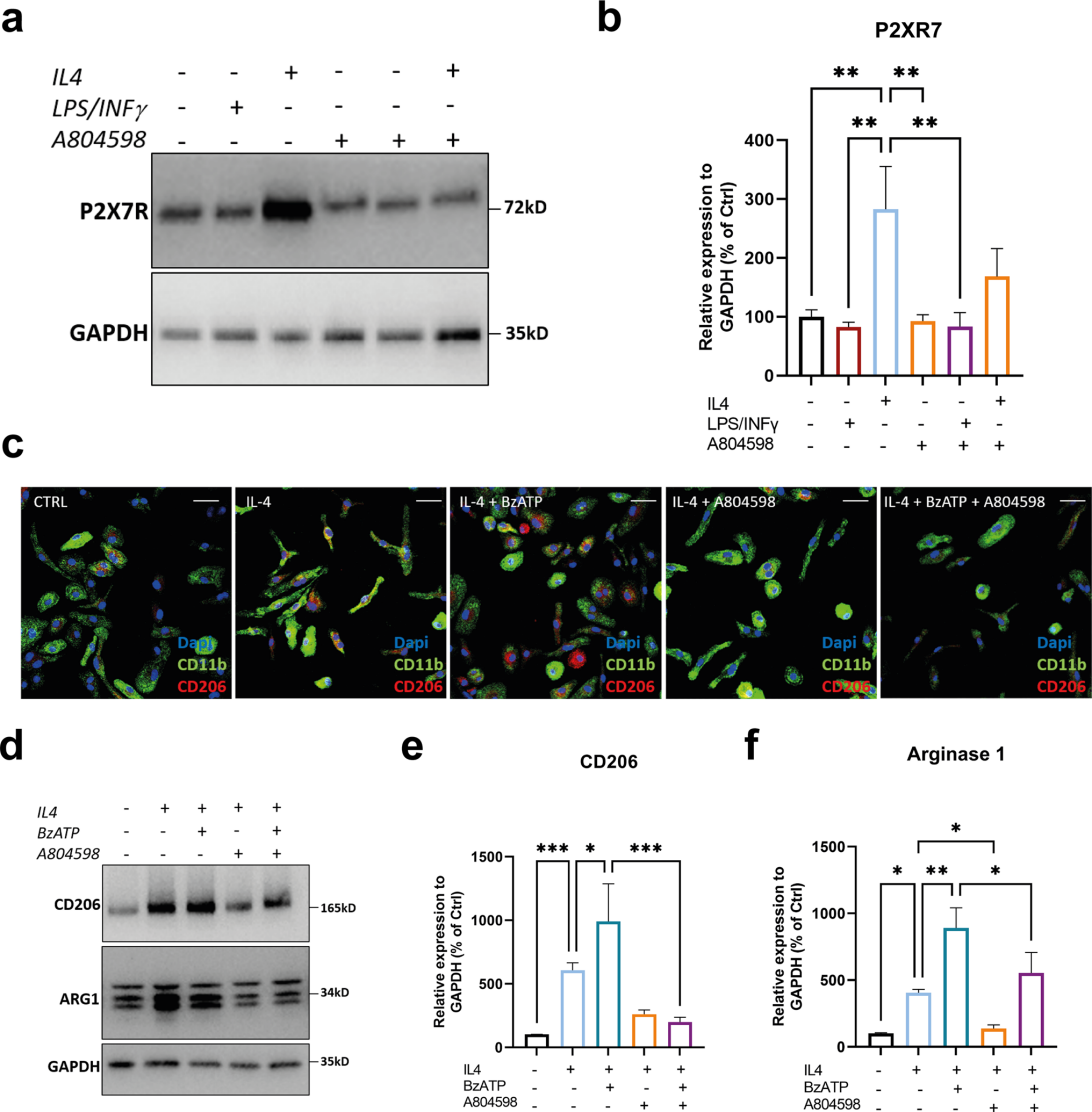
The P2XR7 stimulation enhanced MΦ polarisation towards an M2 anti-inflammatory phenotype. **a**, **b** Representative Immunoblot images and densitometric analysis of P2XR7 levels in ex vivo SOD1G93A-derived MΦ polarised for 24 h to M2 or M1, respectively, with 10 ng/µl IL4 and 1 µg/µl LPS + 20 ng INFγ in presence or absence of 10 μM A804598. The data are reported as percentage of untreated cells (mean ± SEM) of three independent experiments for each group. **P < 0.01 by one-way ANOVA with Tukey’s post-analysis. **c** Representative confocal images showing the immunostaining for CD11b, Cd206 and DAPI in primary SOD1G93A MΦ cultures polarised to M2 for 24 h with 20 ng/µl IL4 or co-exposed to 20 ng/µl IL4 + 100 μM BzATP in presence or absence of 10 μM A804598. Scale bar = 50 µm. **d**–**f** Representative Immunoblot images and densitometric analysis of **d**, **e** CD206 and **d**, **f** Arginase 1 levels in ex vivo SOD1G93A-derived MΦ treated as mentioned in c. The data are reported as percentage of untreated cells (mean ± SEM) of three independent experiments for each group. *P < 0.05, **P < 0.01, ***P < 0.001 by one-way ANOVA with Tukey’s post-analysis

As shown in Fig. 6, P2XR7 was expressed at much higher levels in SOD1G93A M2 cells than in M1 cells and its levels remarkably decreased upon the treatment with the receptor antagonist (Fig. 6a, b). To evaluate the P2XR7 influence on M2-biased MФ, we next cultured spleen–derived SOD1G93A MФ under M2-polarizing conditions in the presence or absence of 100 µM BzATP. We found that the co-administration of IL-4 and BzATP for 24 h heightened the levels of CD206 (Fig. 6c–e) and Arg-1 (Fig. 6d, f) in SOD1G93A MФ compared to IL-4-treated cultures. A804598 antagonism strongly inhibited the induction of SOD1G93A M2 cells, as shown by reduced expression of CD206 (Fig. 6c–e) and Arg-1 (Fig. 6d, f) upon IL-4 or IL-4 + BzATP administration, surmising that the P2XR7 signalling acts synergistically with the MФ maturation pathway to elicit the M2 phenotype.

### The P2XR7 boosting in the hind limb skeletal muscles of SOD1G93A mice preserved the Schwann cell-axon unit and promoted spinal MN survival by decreasing neuroinflammation

**Fig. 7:**
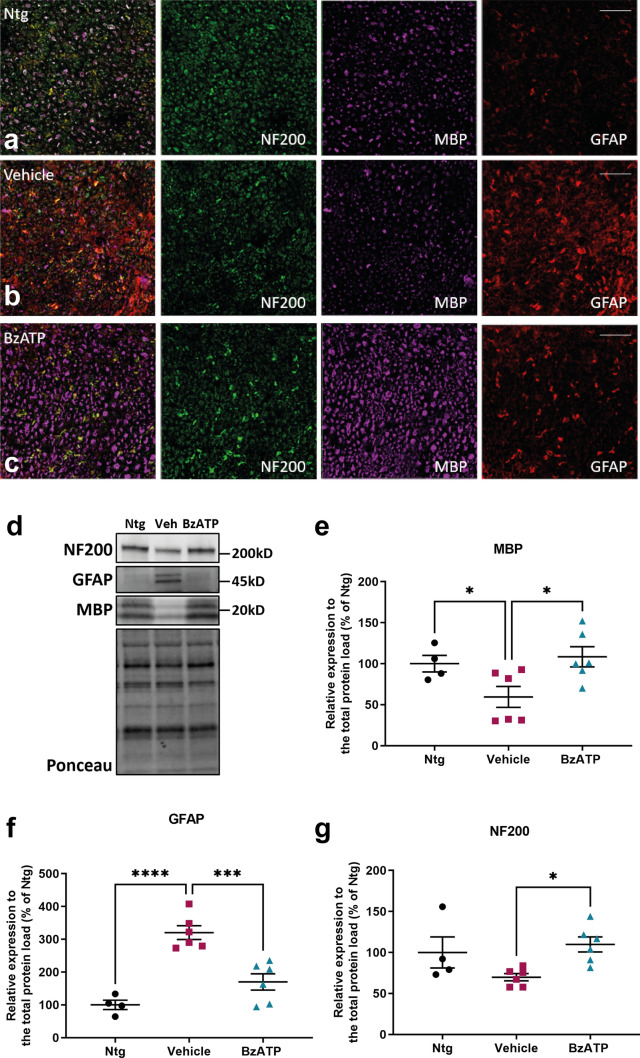
BzATP intramuscular administration preserves the sciatic nerve from axonal degeneration. **a**–**c** Representative confocal images of transverse section of the sciatic nerves of **a** Ntg, **b** BzATP-treated SOD1G93A mice and PBS-treated SOD1G93A mice at 21 weeks showing a higher expression of NFL200 and MBP in **b** BzATP-treated SOD1G93A mice compared to **c** Vehicle mice, which instead show an increase in GFAP protein levels. Images are representative of at least five sections of four independent experiments for each experimental group. Scale bar = 100 µm. **d**–**g** Representative Immunoblot images and densitometric analysis of **d**, **e** MBP, **d**, **f** GFAP and **d**, **g** NF200 in the QC lysates of BzATP- and PBS-treated SOD1G93A mice at **a** 21 weeks of age. The data are reported as percentage of Ntg (mean ± SEM). The independent experiments for each experimental group are scattered on the graph.*P < 0.05 by unpaired *t* test

Given the lower muscle denervation registered in the QC of BzATP- than vehicle-treated SOD1G93A mice during the disease progression, we assessed, by immunoblot and IHC, the protein levels of the Myelin basic protein (MBP) and the Glial fibrillary acidic protein (GFAP), expressed, respectively, by myelinating and non-myelinating Schwann cells [57], within the sciatic nerves of BzATP-treated and vehicle at the symptomatic disease stage. We found a specific increase of MBP (Fig. 7a–e) within the sciatic nerves of BzATP-treated mSOD1 mice, suggesting BzATP-dependent preservation of myelinating motor axons. Conversely, GFAP levels specifically heightened in the sciatic nerves of vehicle-treated mSOD1 mice (Fig. 7a–d, f) while the cytoskeletal protein Neurofilament heavy (NF200) (Fig. 7a–d, g), surmising a derangement of the Schwann cell-axon unit.

We next examined whether the less severe hindlimb pathology and the lower denervation atrophy of hindlimb muscles in BzATP-treated than vehicle-treated mice was reflected in a reduced MN loss within the CNS.

**Fig. 8:**
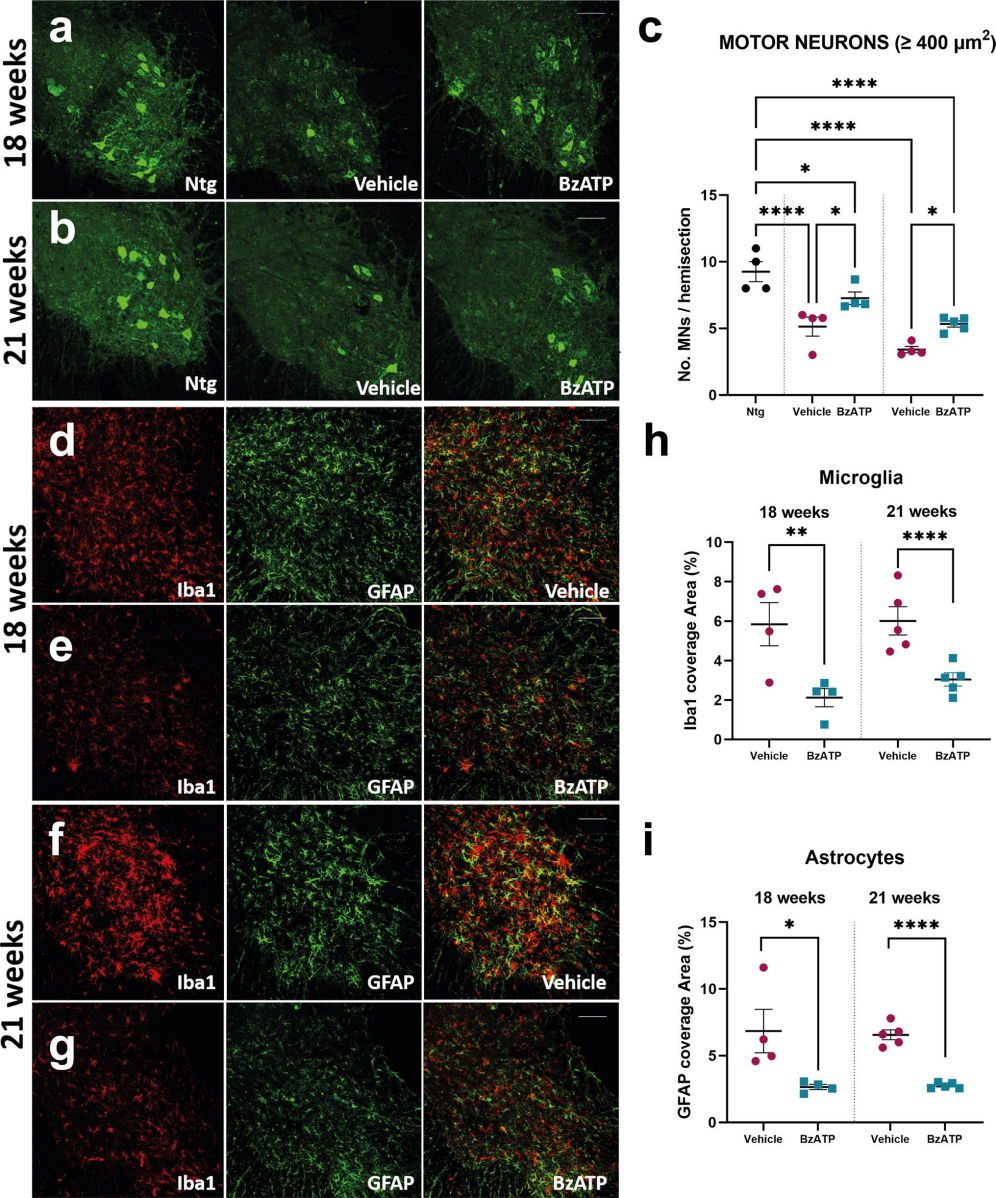
BzATP intramuscular administration reduces motor neuron loss and astrogliosis in the spinal cord of SOD1G93A mice. **a**, **b** Representative confocal images of Chat-immunostained lumbar spinal cord transverse sections of BzATP- and PBS-treated SOD1G93A mice at **a** 18 weeks and **b** 21 weeks of age. Scale bar = 100 µm. **c** The data are expressed as mean ± SEM of MNs (≥ 400 μm^2^) per hemisection. Each symbol in the graph is the average of the quantification of at least ten serial sections (i.e. No. 20 hemisections) for each animal. The independent experiments for each experimental group are scattered on the graph at each time point.*P < 0.05, ****P < 0.0001 by one-way ANOVA with Tukey’s post-analysis. **d**–**g** Representative confocal images showing the staining for Iba1 (red) and GFAP (green) in lumbar spinal cord transverse sections of BzATP- and PBS-treated SOD1G93A mice at **d**, **e** 18 and **f**, **g** 21 weeks of age. Scale bar = 100 µm h, **i** Quantification of the percentage of covered area for **h** Iba1 and **i** GFAP immunostaining in the spinal cord BzATP- and PBS-treated SOD1G93A mice at 18 and 21 weeks of age. The data are expressed as mean ± SEM. Each symbol in the graph is the average of the quantification of at least five sections (i.e. No. 10 hemisections) for each animal. The independent experiments for each experimental group are scattered on the graph at each time point. **P < 0.01, ***P < 0.001, ****P < 0.0001 by unpaired *t* test

Large MNs with a cell body area of ≥ 400 μm^2^ were quantified after ChAT staining in the lumbar spinal cord. Thus, only the large α-MNs, the most vulnerable to cell death in ALS, were quantified [58]. Noteworthy, there was significant protection of MN in the lumbar spinal cord of BzATP-treated mice compared to PBS-treated mice at both the motor disease onset and symptomatic disease stages (Fig. 8a–c). To evaluate if MN protection in the spinal cord was concomitant with reduced inflammation, we next evaluated by IHC the extent of astrogliosis and microgliosis in BzATP-treated mice compared to vehicle-treated mice at the disease motor onset and symptomatic disease stage. Notably, we found a lower GFAP and Ionized calcium-binding adapter molecule 1 (Iba1) immunoreactivity in the ventral horns of the lumbar spinal cord of BzATP-treated mice compared to PBS-treated mice at both the time-points analysed as an index of a reduced inflammatory response (Fig. 8d–i).

### The P2XR7 receptor is differentially activated in the hind limb skeletal muscles of ALS mouse models and it is a muscle-prognostic biomarker in sporadic ALS patients

To evaluate if different ALS-related mutations may impinge on the P2X7R signalling in the hind limb skeletal muscles, we compared the levels of the P2XR7 in different ALS mouse models during the disease progression.

**Fig. 9:**
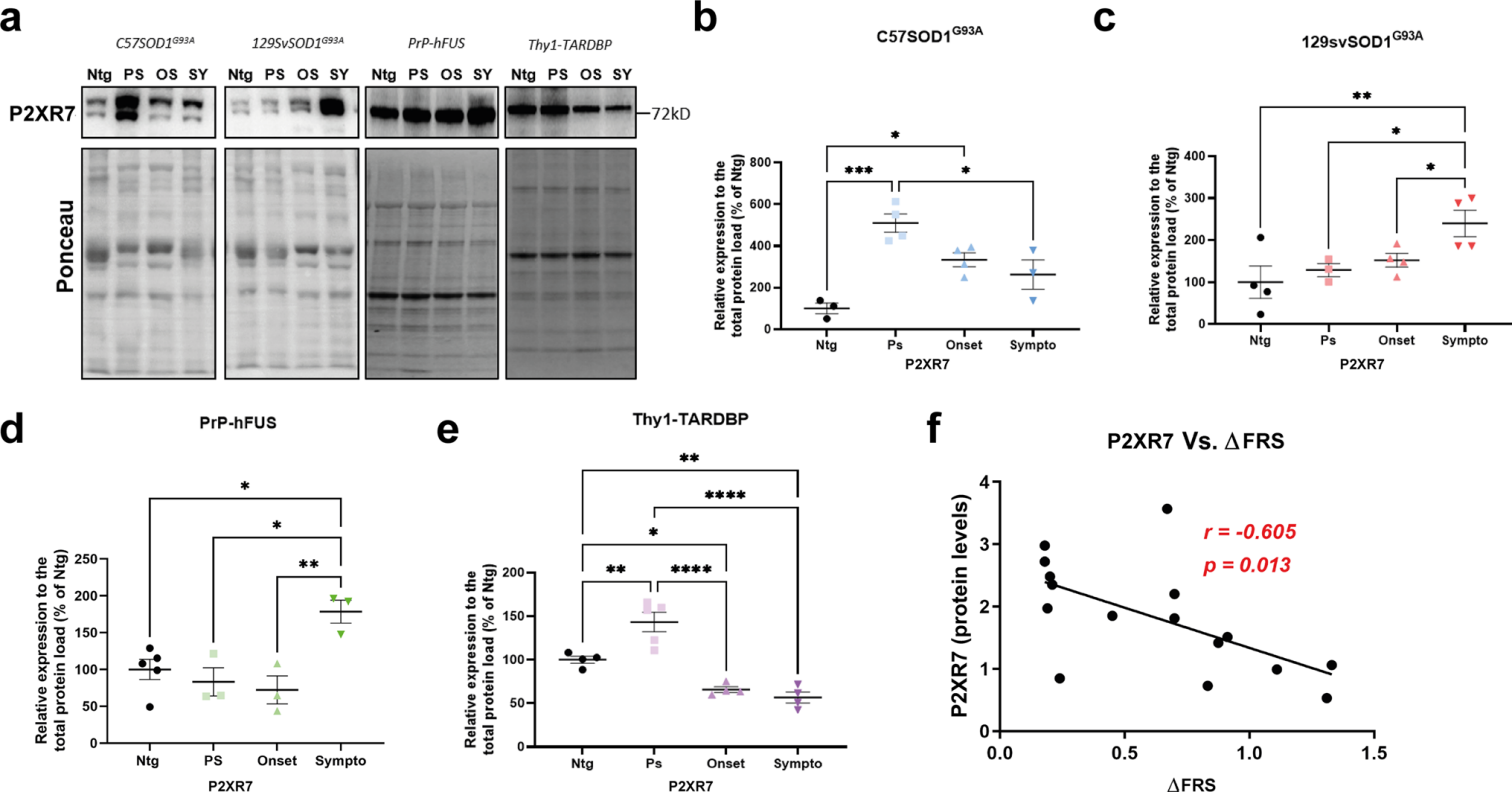
The P2XR7 is differentially modulated in the skeletal muscles of different ALS mouse models and ALS patients with fast and slow disease course. **a**–**e** Representative Immunoblot images and densitometric analysis of P2XR7 in GCM lysates of **a**, **b** C57 SOD1G93A, **a**, **c** 129 Sv SOD1G93A, **a**, **d** PrP-hFUS and **a**, **e** Thy1-TARDBP mice and relative Ntg littermates at presymptomatic (PS), onset (OS) and symptomatic (SY) disease stages. At each disease stage, the data are reported as percentages of the relative Ntg (mean ± SEM) *P < 0.05, **P < 0.01, ***P < 0.001, ****P < 0.0001 by one-way ANOVA with Tukey’s post-analysis. The independent experiments for each mouse model, are scattered on the graph at each time point. **f** Bivariate analysis showing the strength of association between the muscular expression of P2XR7 and the ΔFRS score of ALS patients. The higher is the ΔFRS, the faster is the disease progression. The data were analysed by non-parametric Spearman’s rank correlation

Immunoblot analysis in the GCMs muscles of C57-SOD1G93A mice compared to Ntg mice showed an early and remarked P2XR7 upregulation, which progressively lessened during the later disease stages (Fig. 9a, b). Conversely, the fast progressing 129 Sv-mSOD1 mice with an earlier onset and reduced overall survival than C57-SOD1G93A mice [59] had a delayed and slight upregulation of the P2XR7 receptor at the symptomatic disease stage (Fig. 9a, c). Similarly, the PrP-hFUS mice with an early onset tremor after ~ 4 weeks of age, followed by progressive hindlimb paralysis and death by ~ 12 weeks of age [60], had a late P2XR7 muscle overexpression (Fig. 9a, d). Homozygous Thy1-TARDBP mice, which had an abnormal hindlimb-clasping reflex by two weeks and a twofold decrease in the stride of hindlimbs and forelimbs [61], showed only an early and mild overexpression of P2XR7 in the skeletal muscles, which is followed by an abrupt downregulation at the later disease stages compared to Ntg mice (Fig. 9a, e).

Skeletal muscle is one of the most severely affected tissue by ALS and it is easily accessible to biopsy even during the disease progression [62]. However, there are still few developments in clinical muscle diagnosis [63, 64].

Here, we undertook a pilot study into clinical pre-validation in muscles biopsies of the left *Vastus* *Lateralis* muscle from No. 19 age-matched ALS patients with fast and slow disease progression, identified by the Δ Functional Rating Scale (ΔFRS) (Supplementary Table 2) [40], to evaluate a correlation among P2XR7 protein muscle levels and the rate of disease progression.

Immunoblot analysis showed an inverse relationship between the expression level of the purinergic receptor in the skeletal muscle and the speed of the disease progression of ALS patients (Fig. 9 f; Supplementary Fig. 6).

## Discussion

In this study, we further explored the involvement of the P2XR7 in ALS by virtue of in vitro and in vivo experimental paradigms on mSOD1 ALS mouse models.

We found that the intramuscular boosting of P2XR7 through BzATP resulted in a remarked improvement of the motor function of SOD1G93A mice. This was associated with a straight BzATP influence on the proliferation and differentiation of SCs and a P2XR7-mediated effect on macrophage recruitment and polarisation within the skeletal muscles of transgenic ALS mice. The beneficial effect on skeletal muscles then spread retrogradely to the motor axons in the sciatic nerves and MNs within the spinal cord.

We recently showed that the systemic BzATP administration in SOD1G93A mice just before the onset of a pathology improved the innervation and metabolism of myofibres, moreover eliciting myogenesis, thus preventing the denervation atrophy of skeletal muscles in SOD1G93A mice [37].

To make the treatment potentially translatable into the clinical practice, we intramuscularly injected BzATP within the hindlimb skeletal muscles of ALS transgenic mice at the presymptomatic disease stage. This approach improved motor activity in SOD1G93A mice with a postponement of symptom onset compared to controls. As in the previous study, we found that BzATP administration preserved the denervation atrophy of hindlimb skeletal muscles, which was associated with increased muscle fibre size due to an enhanced expression of pro-differentiating factors, MyoD and MyoG, at the disease onset.

### Boosting the P2XR7 signalling enhances myogensis through the activation of ERK ½ pathway

Mature myotubes present higher ATP release, P2 receptor surface expression and activity and ATP-hydrolysing enzyme expression than undifferentiated myoblasts [65]. Among P2XRs, P2XR7 protein expression is low in quiescent myoblasts; it increases in proliferating myoblasts and is highly represented in myotubes. In myoblasts, P2XR7 stimulates proliferation, which is enhanced by BzATP. P2XR7 blockade, via BzATP antagonism prevents myotubes formation, suggesting a role in full myoblast differentiation, although the specific molecular mechanisms still need to be clarified [66].

We previously corroborated this evidence in ex vivo myofibres from SOD1G93A mice showing how BzATP administration compensated for SOD1G93A SCs defective proliferation by increasing the number of Pax7^+^ cells per fibres and promoting their differentiation [37]. In the present study, we further implemented these observations showing how SCs derived from BzATP-treated muscles and cultured ex vivo have an improved aptitude to proliferation, suggesting an imprinted memory resulting from environmental cues. Indeed, the direct BzATP induction of the P2XR7 signalling in primary SOD1G93A SC cultures remarkably boosted their proliferation and differentiation, which are fully inhibited by the BzATP antagonism.

Albeit P2XR7 is classically associated with the induction of inflammatory pathways ranging from the leucine-rich repeat (LRR)-containing protein 3 (NLRP3)-inflammasome activation to the NF-kB transcription factor induction, studies on nucleotide receptors signalling in the CNS revealed that P2XR7 activates signalling cascades characteristic of trophic factors [49, 50].

In primary cultures of cerebellar granule neurons, the inhibition of GSK3 activity induced by P2XR7 receptors promotes neuroprotection [67, 68]. The trophic activity of the P2XR7 receptor in granule neurons also accounts for coupling to extracellular signal-regulated kinases ERK½-MAPKs [69]. In this case, the effect is directly related to P2XR7-stimulated calcium signalling and the activation of cAMP response element-binding protein (CREB) [70]. Alternatively, nucleotides acting at P2XR7 can couple to canonical PI3K/Akt signalling, which can promote the survival and proliferation of cortical astrocytes after mechanical strain caused injury [71].

Among these pathways, we showed that the induction of P2XR7 through BzATP specifically elicited the ERK1/2 phosphorylation in the skeletal muscles of SOD1G93A mice. Besides, BzATP administration to primary SOD1G93A SC cultures increased ERK1/2 phosphorylation and this effect is fully abolished following the P2XR7 antagonism. Our results are in line with the data in literature indicating that the ERK1/2 pathway is involved in promoting the activation of SCs and myogenesis [72–74].

### The P2XR7 signalling affects the MФ differentiation towards an M2-biased phenotype

The inflammatory response is coupled temporally and spatially to myogenesis, and it has a central role in bridging initial muscle injury responses and muscle reparation [53]. Complete muscle tissue repair is strongly dependent on the timely recruitment of blood monocytes that enter the damaged tissue and differentiate into distinct MФ subtypes [52, 75, 76]. Pro-inflammatory MФ accumulate first in the injured tissue area to phagocytose debris and stimulate SCs, subsequently converting to anti-inflammatory MФ that support the formation and growth of new myofibres [77–79]. In vivo studies in CD11b-diphtheria toxin receptor (CD11b-DTR) transgenic mice or following clodronate liposome or Etoposide administration have demonstrated that MФ depletion severely impairs skeletal muscle regeneration with the formation of smaller myofibres [79–83]. Besides, MФ response is pivotal to control the cell fate and, in turn, SC regenerative potential in chronic muscle diseases. Indeed, the exhaustion of MФ caused an exacerbated dystrophic phenotype in *mdx* mice with a transient and local MФ depletion [84].

P2X7R was first described MФ and later identified in immune cell types belonging to innate and adaptive immunity [29]. This facet has never been addressed in ALS, albeit the peripheral immune response has an active role in the disease progression of ALS mice in the CNS and PNS [85–89].

It was previously found that BzATP administration to SOD1G93A ex vivo cultures of primary microglia elicits either M1 or M2 phenotypes depending on the persistence of activation, surmising a pivotal role of the P2XR7 signalling in defining the inflammatory fingerprint of myeloid cells [31]. In keeping with this, the recent data suggested that the purinergic signal is essential to regulate MФ maturation to M2-like phenotype [54, 90] and Torre-Minguela et al. [91] reported that ATP-dependent P2XR7 stimulation in MФ is able to release potent anti-inflammatory proteins suggesting a potential role for P2XR7 during resolution of the inflammation.

In the present study, we found that the BzATP-P2XR7 signalling influenced the peripheral immune response within the skeletal muscle of SOD1G93A mice during myogenesis. Intramuscular BzATP administration at the presymptomatic stage in transgenic mice promoted an enrichment of M2-MФ within the skeletal muscles at the disease onset, which spread to the symptomatic phase, albeit to a lesser extent. MФ polarisation at the disease onset coincided with the downregulation of M1-MФ– derived factors, IGF1 and TNFα, in favour of the increased levels of IL-10, expressed by M2‑biased MФ to attenuate the inflammatory response and elicit the differentiation of SCs to myocytes.

We established the direct influence of P2XR7 on MФ-M2 polarisation by showing higher receptor levels in SOD1G93A M2-polarised MФ than SOD1G93A- M1-polarised MФ. In addition, we found that the co-administration of IL-4 and BzATP to SOD1G93A-derived MФ cultures enhanced the M2 profile associated with the increased expression of Arg1 and CD206. Conversely, in vitro polarisation shift of MФ towards M2 phenotype was strongly suppressed following P2X7R antagonism.

Our data suggest that BzATP acts directly on SCs by enhancing their proliferation and differentiation and indirectly promoted myogenesis by influencing MФ and their shifting phenotype, which is an essential step to promote muscle formation and growth of new myofibres.

### Preservation of skeletal muscle transduced retrogradely along the motor unit of SOD1G93A mice

Skeletal muscle is a source of anabolic signals that influence neuron survival, axonal growth and maintenance of synaptic connections [23]. Genetic mouse models showed that the restricted expression of mSOD1 to MNs did not trigger the ALS pathology [92]. Conversely, mSOD1 expression in skeletal muscle elicited muscle atrophy, decreased muscle strength, reduced spinal cord mass, triggered late MN loss and shortened lifespan [19]. Another study reported that muscle-restricted expression of the human mutant SOD1 gene causes motor neuron degeneration in old transgenic mice [20]. Moreover, muscle-selective alterations in mitochondrial function occur in SOD1G93A mice before disease onset and may initiate the destruction of the neuromuscular junction, followed by distal axonopathy, astrocytosis in the spinal cord and mild motor neuron loss [15, 23, 93]. This evidence indicates an intrinsic muscle pathology directly affecting MNs and, consequently, the quality of connections of the neuromuscular units.

In this context, it was described as the altered activity of SCs following a muscle insult is transmitted along the body by secretion of different cytokines/factors not only to other SCs in distant muscles but also to the immune system and endothelium [94, 95]. Therefore, signals coming from new forming myofibres and SCs implied in the process could be crucial in the context of preventing MN loss, especially considering that SC secretory function is essential for neuromuscular junction (NMJ) preservation [23, 96, 97].

Based on these data, we extended our investigation beyond the skeletal muscles to evaluate if muscle preservation was reflected in the protection of the whole motor unit.

In the sciatic nerves of SOD1G93A mice, BzATP intramuscular treatment resulted in the preservation of motor axons myelination, which reflected in the maintenance of the Schwann cell-axon unit. Besides, we found a reduced MN loss in the spinal cord associated with a decreased astrogliosis across the disease progression.

Our results indirectly support the evidence indicating that retrograde neurodegeneration of MNs could be an integral part of ALS pathogenesis in a target deprivation type scenario where primary pathology in skeletal muscle, including distal NMJ dismantling, could set up the target deprivation of MNs [98, 99]. Indeed, we established that improving the skeletal muscle is sufficient to partially preserve the whole motor unit, hinting that MN degeneration in SOD1G93A transgenic mice might follow muscle disease and be a form of retrograde dying-back degeneration with similarities to human ALS [23, 100].

Based on this scenario, P2XR7 agonism may represent an effective therapeutic adjunct in ALS, considering it is transversally induced in the skeletal muscle of different ALS mouse models during the disease progression. Albeit it is arduous to draw firm conclusions, it is possible to hypothesise that the early and remarked upregulation of P2XR7 in the slow-progressing C57-SOD1G93A mice could be an attempt by the system to counteract muscle atrophy. Conversely, the lower or late overexpression in rapidly progressing Thy1-TARDBP, 129 Sv-SOD1G93A and PrP-hFUS mice might be representative of a more severe disease progression. This issue is extendable to ALS patients where the P2XR7 levels in the skeletal muscles correlated inversely with the severity of the disease.

## Conclusions

We showed that the intramuscular boosting of the P2XR7 signalling in the skeletal muscles of SOD1G93A mice improved the motor performance by decreasing the denervation atrophy, preserving motor axons and mitigating astrogliosis and MN loss.

Making a compendium with the data previously illustrated [37], overall, we established a multitarget activity of the P2XR7 signalling within the skeletal muscle of transgenic ALS mice. The triggering of P2XR7 enhanced the metabolism of muscle fibres, preserved the NMJ morphology and promoted myogenesis. The latter is induced through the direct influence of BzATP on SCs and its indirect action on MФ polarisation towards an anti-inflammatory and pro-regenerative phenotype.

Previous results showed that the systemic pharmacological antagonism of P2XR7 was ineffective in counteracting the disease progression or slightly improved the survival of SOD1G93A mice [24, 25]. This is possibly due to the multifaceted role of the P2XR7 axis, ranging from the activation of pro-survival signalling to the induction of inflammation based on the context of activation [26–30].

The many therapeutic failures have reinforced the idea that ALS is a multi-factorial and multi-systemic disease in which alterations in structural, physiological and metabolic parameters in MNs, glia and muscle act synergistically to exacerbate the disease. Thus, to be effective, therapeutic approaches should target multiple mechanisms and various cells/tissues [22, 101]. Here, we demonstrated that the contingent boosting of P2XR7 within the skeletal muscles could be a groundbreaking multisystem therapeutic strategy in ALS to be used in conjunction with CNS-targeted drugs to enhance the effectiveness of potential clinical treatments.

## Supplementary Information

Below is the link to the electronic supplementary material.

Supplementary file1 (DOCX 15 KB)

Supplementary file2 (TIF 9205 KB) Supplementary Fig. 1 The P2XR7 is differentially expressed in the hindlimb muscles of SOD1G93A mice. a, b Representative Immunoblot images and densitometric analysis of P2XR7 in TA, QC and GCM lysates of C57 SOD1G93A mice at 12 weeks of age. Data are reported as fold change P2XR7 variation compared to Ntg littermates (mean ±SEM). The independent experiments for each experimental group are scattered on the graph. *P <0.05, ****P<0.0001 by unpaired *t* test

Supplementary file3 (TIF 2286 KB) Supplementary Fig. 2 BzATP intramuscular administration increases the levels of myofibre pro-differentiation factors in the skeletal muscle of SOD1G93A mice. a–d Representative Immunoblot images and densitometric analysis of (a, b) Pax7, (a, c) MyoD and (a, d) MyoG in the QC lysates of BzATP- and PBS-treated SOD1G93A mice at (a) 18 weeks of age. Data are reported as percentage of Ntg (mean ±SEM). The independent experiments for each experimental group are scattered on the graph. *P <0.05 by unpaired *t* test.

Supplementary file4 (TIF 13355 KB) Supplementary Fig. 3 BzATP intramuscular administration increases the proliferation index of ex vivo SOD1G93A mice–derived satellite cells. a Representative optical images of ex vivo SOD1G93A SCs derived from BzATP-treated or untreated hindlimb muscles and cultured four days in growth medium (GM4). Scale bar = 100 µm b At GM4, SOD1G93A SCs derived from BzATP-treated hindlimb muscles show a higher proliferation index than control as further confirmed by higher expression of c, d PAX7; c, e MYOD; and c, f P2XR7. Data are reported as mean ± SEM of three independent experiments for each group. *P <0.05 by unpaired *t* test.

Supplementary file5 (TIF 1357 KB) Supplementary Fig. 4 Muscle wasting was calculated by measuring of the Quadriceps muscle weight of BzATP- and PBS-treated SOD1G93A mice compared to Ntg littermates. Data are presented as mean ± SEM. The independent experiments are scattered on the graph for each experimental group. *P<0.05 by unpaired *t* test.

Supplementary file6 (TIF 3988 KB) Supplementary Fig. 5 a, b P2XR7 heightened its expression on the cell membrane upon BzATP treatment. Representative confocal images of satellite cells showing an increased colocalisation between P2XR7 and wheat germ agglutinin (WGA) upon treatment with BzATP for 15’. Scale bar = 100 µm.

Supplementary file7 (DOCX 19 KB)

Supplementary file8 (TIF 17812 KB) Supplementary Fig. 6 The P2XR7 levels are higher in muscle biopsies of slow progressing than fast progressing ALS patients. Representative Immunoblot images of P2XR7 in the Vastus Lateralis muscle lysates of fast and slow progressing ALS patients (see Supplementary Table 2). Immunoreactivity was normalised to the total amount of protein detected by the Stain-Free membrane activation system (BioRad). Given the analysis of two different membranes, the P2XR7 levels were analysed as follows: i) an internal standard (IS) representing the mix of all the samples in the experiment was loaded on each gel; ii) membranes were acquired at the same time; iii) the immunoreactivity of each sample was further normalised to the immunoreactivity of the IS.

## Data Availability

Data, materials and software information supporting the conclusions of this article are included within the article and its additional files.

## Abbreviations

MN: Motor neuron
mSOD1: Mutant superoxide dismutase 1
SC: Satellite cell
MΦ: Macrophage
BzATP: 2′(3′)-*O*‐(4-Benzoylbenzoyl) adenosine 5-triphosphate
QC: Quadriceps
TA: Tibialis anterior
GCM: Gastrocnemius medialis
NMJ: Neuromuscular junction
