# Supplementary material for: Contingent intramuscular boosting of P2XR7 axis improves motor function in transgenic ALS mice
Source: Cell Mol Life Sci. 2021 Dec 22;79(1):7. doi: 10.1007/s00018-021-04070-8 (PMC8695421; doi:10.1007/s00018-021-04070-8)
Supplement: Supplementary file 1 — Supplementary file1 (DOCX 15 KB) [file 18_2021_4070_MOESM1_ESM.docx]

|  | **ID** | **Genotype** | **BodyWeight (gr)** | **Treatment** |
| --- | --- | --- | --- | --- |
| **Mice sacrificed at 21 weeks** | 800 | *SOD1G93A* | **19.5** | **BzATP** |
|  | 805 | *SOD1G93A* | **20.8** | **BzATP** |
|  | 807 | *SOD1G93A* | **21.5** | **Vehicle** |
|  | 843 | *SOD1G93A* | **17.3** | **Vehicle** |
|  | 854 | *SOD1G93A* | **19.4** | **BzATP** |
|  | 849 | *SOD1G93A* | **20.5** | **Vehicle** |
|  | 850 | *SOD1G93A* | **21.2** | **BzATP** |
|  | 875 | *SOD1G93A* | **17.9** | **BzATP** |
|  | 879 | *SOD1G93A* | **21.1** | **Vehicle** |
|  | 881 | *SOD1G93A* | **22.3** | **BzATP** |
|  | 502 | *SOD1G93A* | **17.7** | **BzATP** |
|  | 486 | *SOD1G93A* | **17.6** | **Vehicle** |
|  | 511 | *SOD1G93A* | **21.7** | **BzATP** |
|  | 507 | *SOD1G93A* | **19.4** | **Vehicle** |
|  | 506 | *SOD1G93A* | **20.2** | **BzATP** |
|  | 432 | *SOD1G93A* | **22.7** | **Vehicle** |
|  | 414 | *SOD1G93A* | **19.8** | **Vehicle** |
|  | 413 | *SOD1G93A* | **18.8** | **BzATP** |
|  | 412 | *SOD1G93A* | **20** | **Vehicle** |
|  | 411 | *SOD1G93A* | **21.7** | **BzATP** |
|  | 373 | *SOD1G93A* | **21.9** | **Vehicle** |
|  | 371 | *SOD1G93A* | **22.2** | **BzATP** |
| **Mice sacrificed at 18 weeks** | 1303 | *SOD1G93A* | **18,2** | **BzATP** |
|  | 1305 | *SOD1G93A* | **21,7** | **Vehicle** |
|  | 1276 | *SOD1G93A* | **20,6** | **Vehicle** |
|  | 1175 | *SOD1G93A* | **21** | **BzATP** |
|  | 1176 | *SOD1G93A* | **17,5** | **Vehicle** |
|  | 1226 | *SOD1G93A* | **22.2** | **BzATP** |
|  | 1179 | *SOD1G93A* | **18** | **Vehicle** |
|  | 1229 | *SOD1G93A* | **19.8** | **BzATP** |
|  | 1230 | *SOD1G93A* | **19.6** | **Vehicle** |
| **Mice sacrificed at 13 weeks** | 1076 | *SOD1G93A* | **20,8** | **BzATP** |
|  | 1071 | *SOD1G93A* | **19,4** | **Vehicle** |
|  | 1066 | *SOD1G93A* | **17,9** | **BzATP** |
|  | 1064 | *SOD1G93A* | **18,8** | **BzATP** |
|  | 1063 | *SOD1G93A* | **19,8** | **Vehicle** |
|  | 1050 | *SOD1G93A* | **19,8** | **Vehicle** |
|  | 1048 | *SOD1G93A* | **20,1** | **BzATP** |
|  | 1139 | *SOD1G93A* | **20,5** | **BzATP** |
|  | 1141 | *SOD1G93A* | **21,9** | **Vehicle** |
|  | 1142 | *SOD1G93A* | **19,8** | **Vehicle** |

***Supplementary Table 1*** *Mice* *bodyweight at the time of assignment into experimental groups*
