# Supplementary figures and images for: Contingent intramuscular boosting of P2XR7 axis improves motor function in transgenic ALS mice
Source: Cell Mol Life Sci. 2021 Dec 22;79(1):7. doi: 10.1007/s00018-021-04070-8 (PMC8695421; doi:10.1007/s00018-021-04070-8)

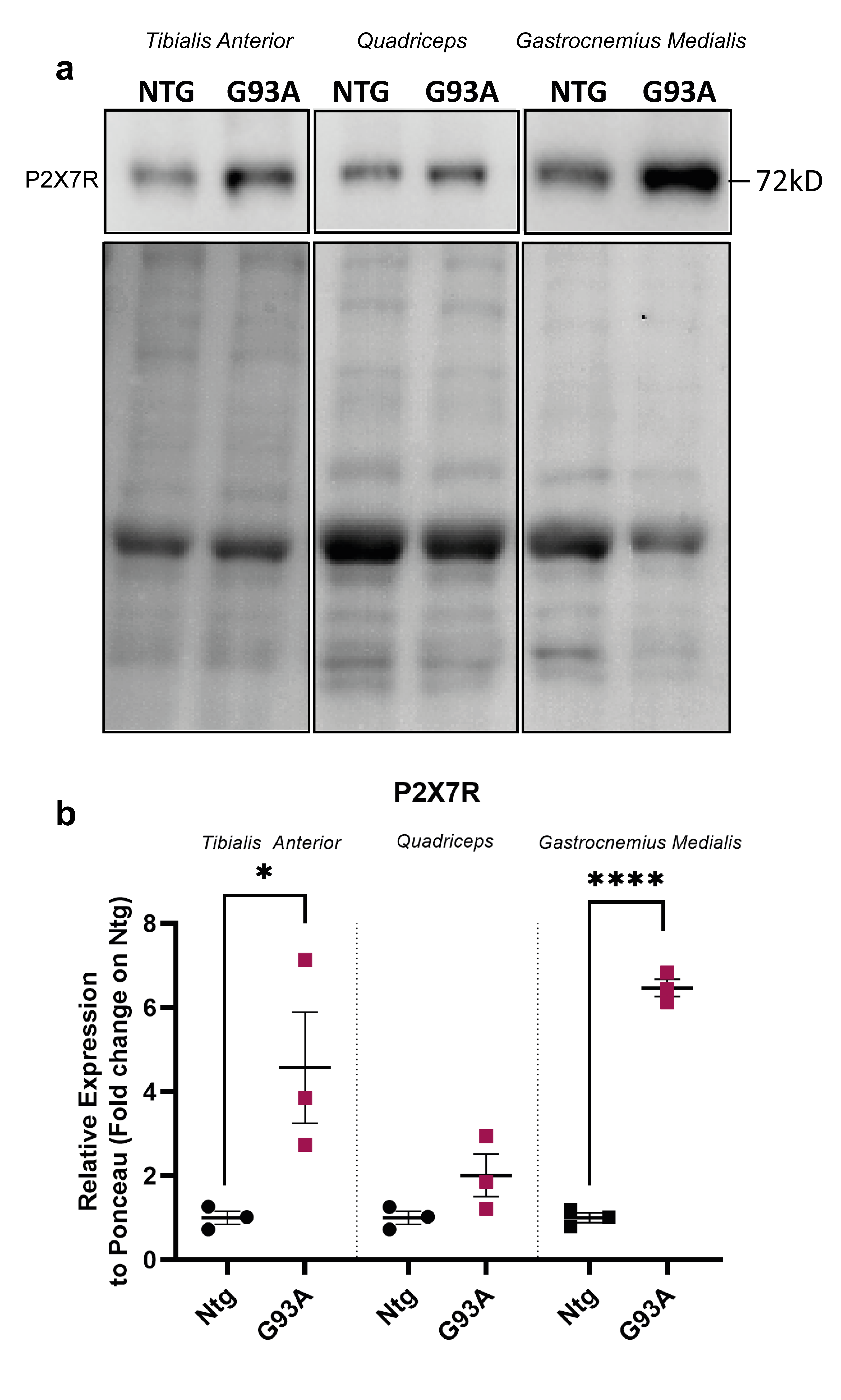

Supplement: Supplementary file 2 — Supplementary file2 (TIF 9205 KB) Supplementary Fig. 1 The P2XR7 is differentially expressed in the hindlimb muscles of SOD1G93A mice. a, b Representative Immunoblot images and densitometric analysis of P2XR7 in TA, QC and GCM lysates of C57 SOD1G93A mice at 12 weeks of age. Data are reported as fold change P2XR7 variation compared to Ntg littermates (mean ±SEM). The independent experiments for each experimental group are scattered on the graph. *P <0.05, ****P<0.0001 by unpaired t test [file 18_2021_4070_MOESM2_ESM.tif]

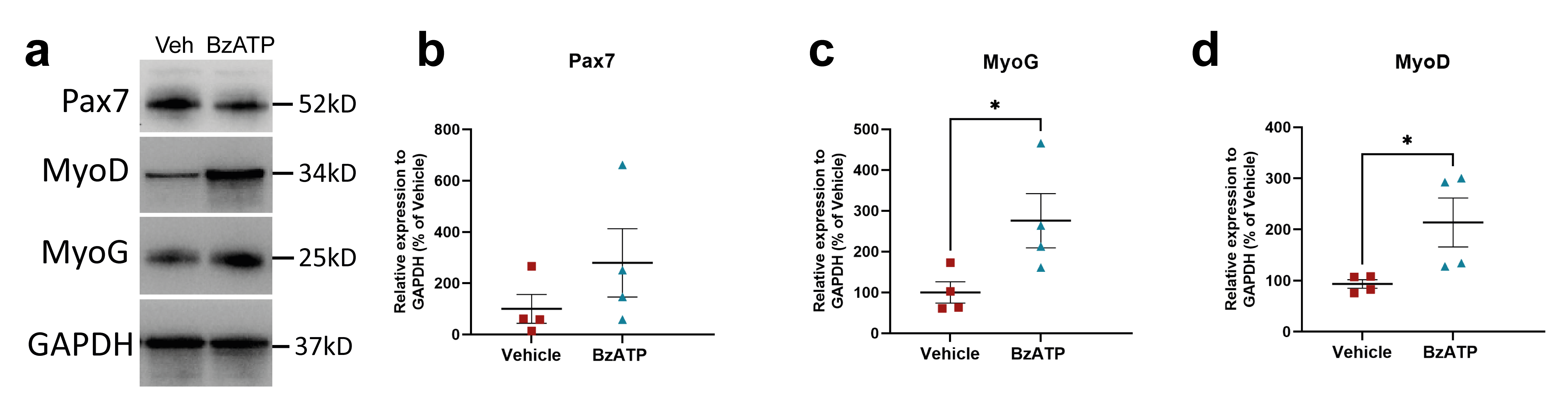

Supplement: Supplementary file 3 — Supplementary file3 (TIF 2286 KB) Supplementary Fig. 2 BzATP intramuscular administration increases the levels of myofibre pro-differentiation factors in the skeletal muscle of SOD1G93A mice. a–d Representative Immunoblot images and densitometric analysis of (a, b) Pax7, (a, c) MyoD and (a, d) MyoG in the QC lysates of BzATP- and PBS-treated SOD1G93A mice at (a) 18 weeks of age. Data are reported as percentage of Ntg (mean ±SEM). The independent experiments for each experimental group are scattered on the graph. *P <0.05 by unpaired t test. [file 18_2021_4070_MOESM3_ESM.tif]

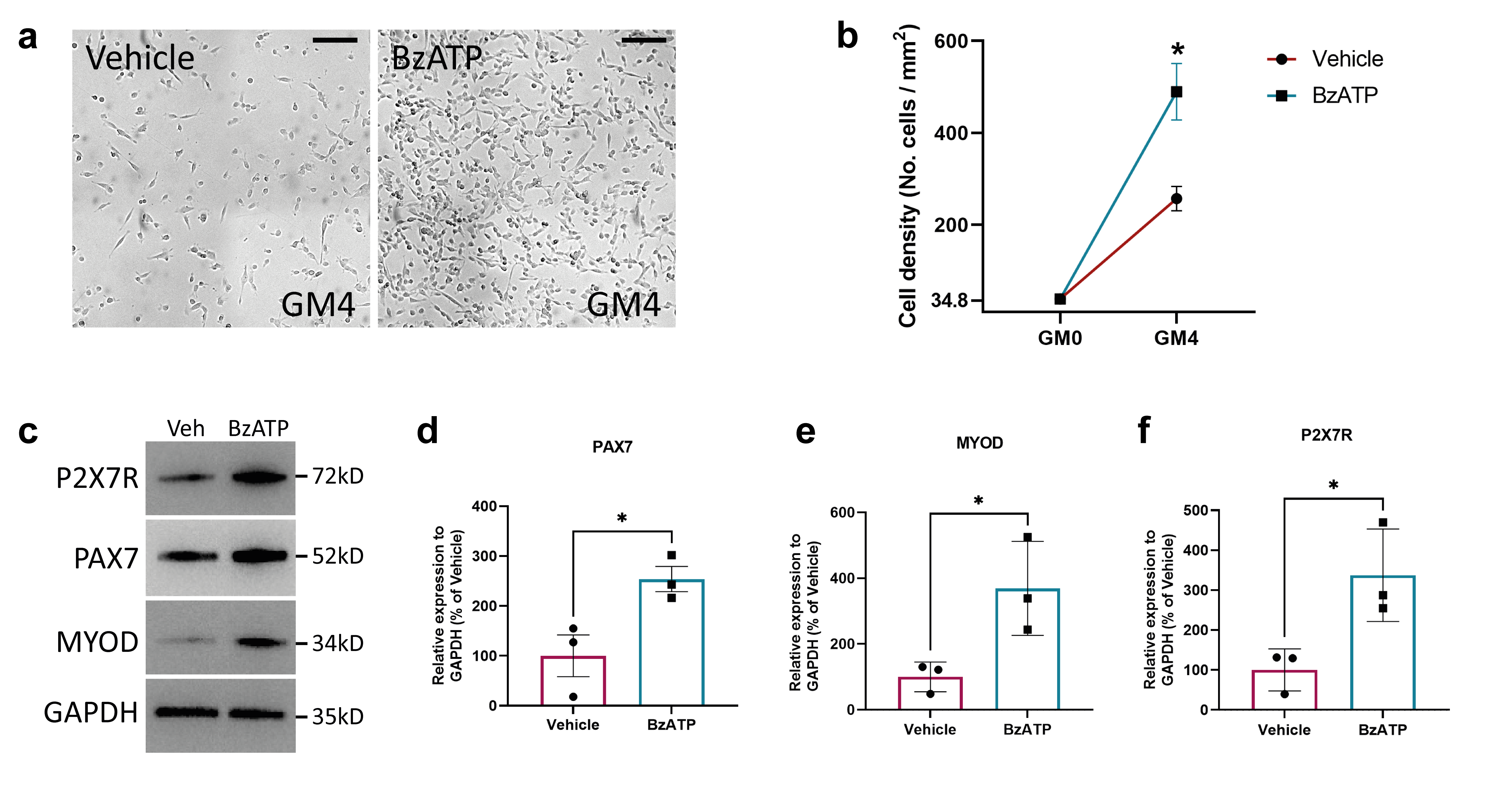

Supplement: Supplementary file 4 — Supplementary file4 (TIF 13355 KB) Supplementary Fig. 3 BzATP intramuscular administration increases the proliferation index of ex vivo SOD1G93A mice–derived satellite cells. a Representative optical images of ex vivo SOD1G93A SCs derived from BzATP-treated or untreated hindlimb muscles and cultured four days in growth medium (GM4). Scale bar = 100 µm b At GM4, SOD1G93A SCs derived from BzATP-treated hindlimb muscles show a higher proliferation index than control as further confirmed by higher expression of c, d PAX7; c, e MYOD; and c, f P2XR7. Data are reported as mean ± SEM of three independent experiments for each group. *P <0.05 by unpaired t test. [file 18_2021_4070_MOESM4_ESM.tif]

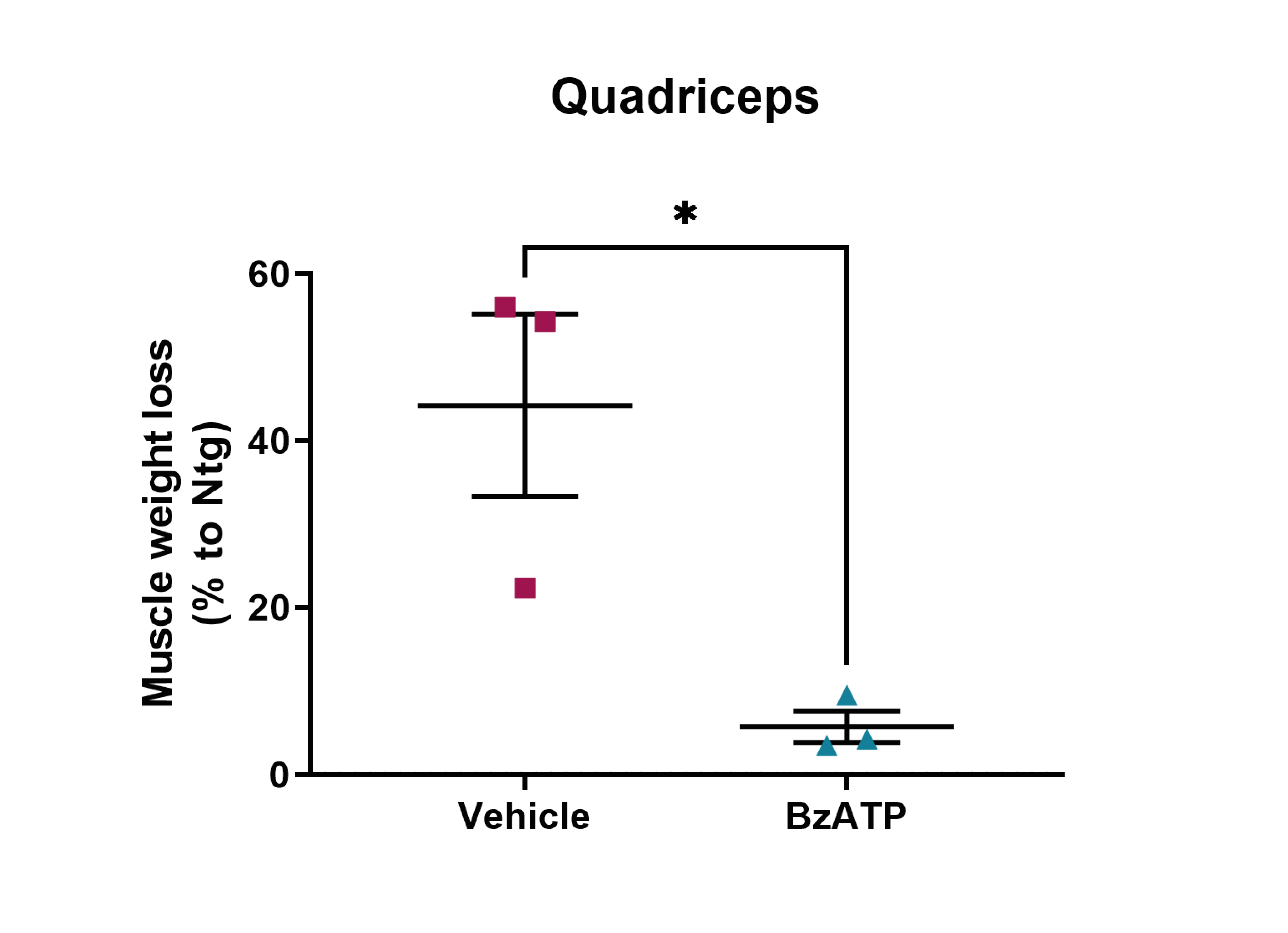

Supplement: Supplementary file 5 — Supplementary file5 (TIF 1357 KB) Supplementary Fig. 4 Muscle wasting was calculated by measuring of the Quadriceps muscle weight of BzATP- and PBS-treated SOD1G93A mice compared to Ntg littermates. Data are presented as mean ± SEM. The independent experiments are scattered on the graph for each experimental group. *P<0.05 by unpaired t test. [file 18_2021_4070_MOESM5_ESM.tif]

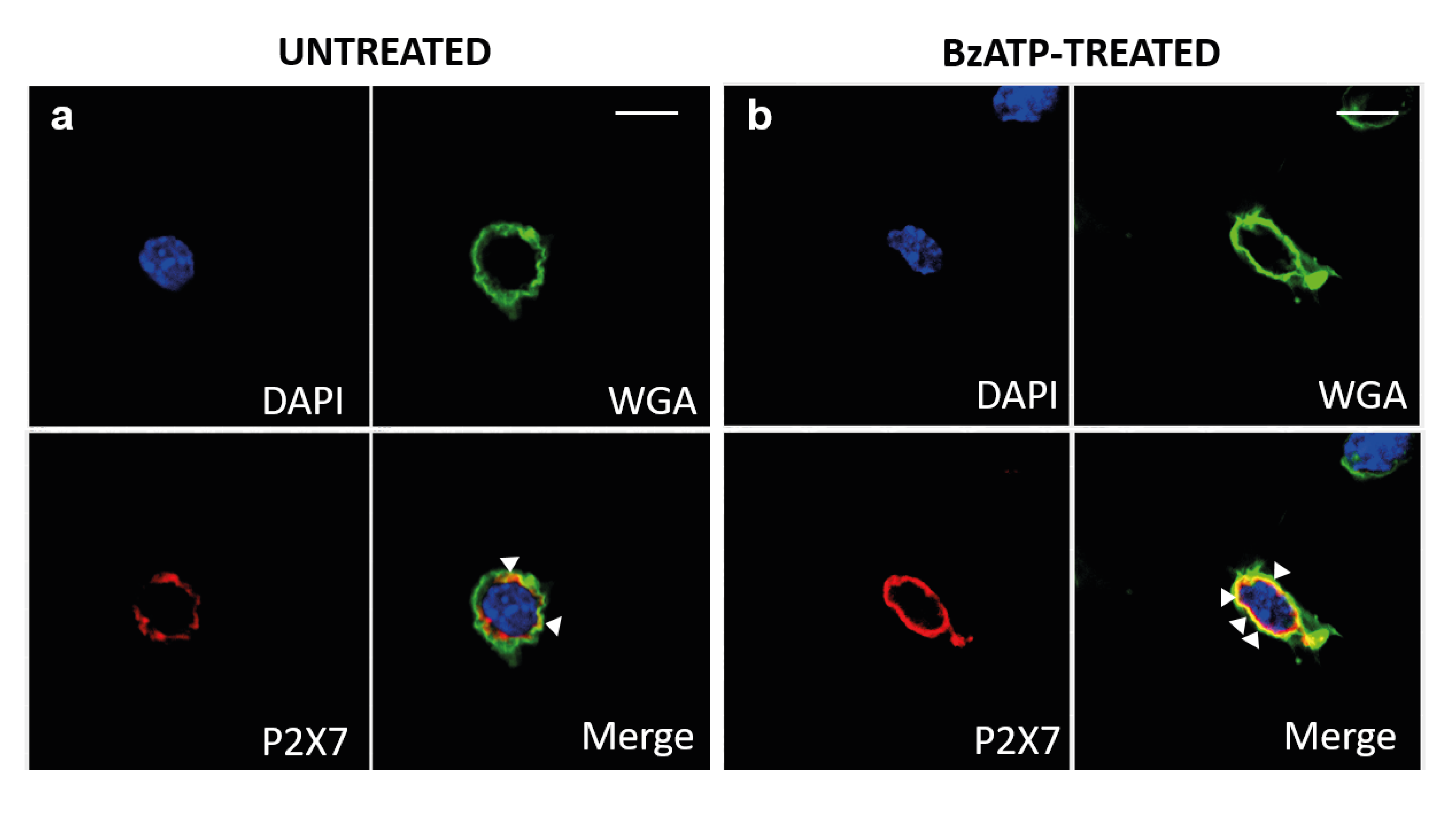

Supplement: Supplementary file 6 — Supplementary file6 (TIF 3988 KB) Supplementary Fig. 5 a, b P2XR7 heightened its expression on the cell membrane upon BzATP treatment. Representative confocal images of satellite cells showing an increased colocalisation between P2XR7 and wheat germ agglutinin (WGA) upon treatment with BzATP for 15’. Scale bar = 100 µm. [file 18_2021_4070_MOESM6_ESM.tif]

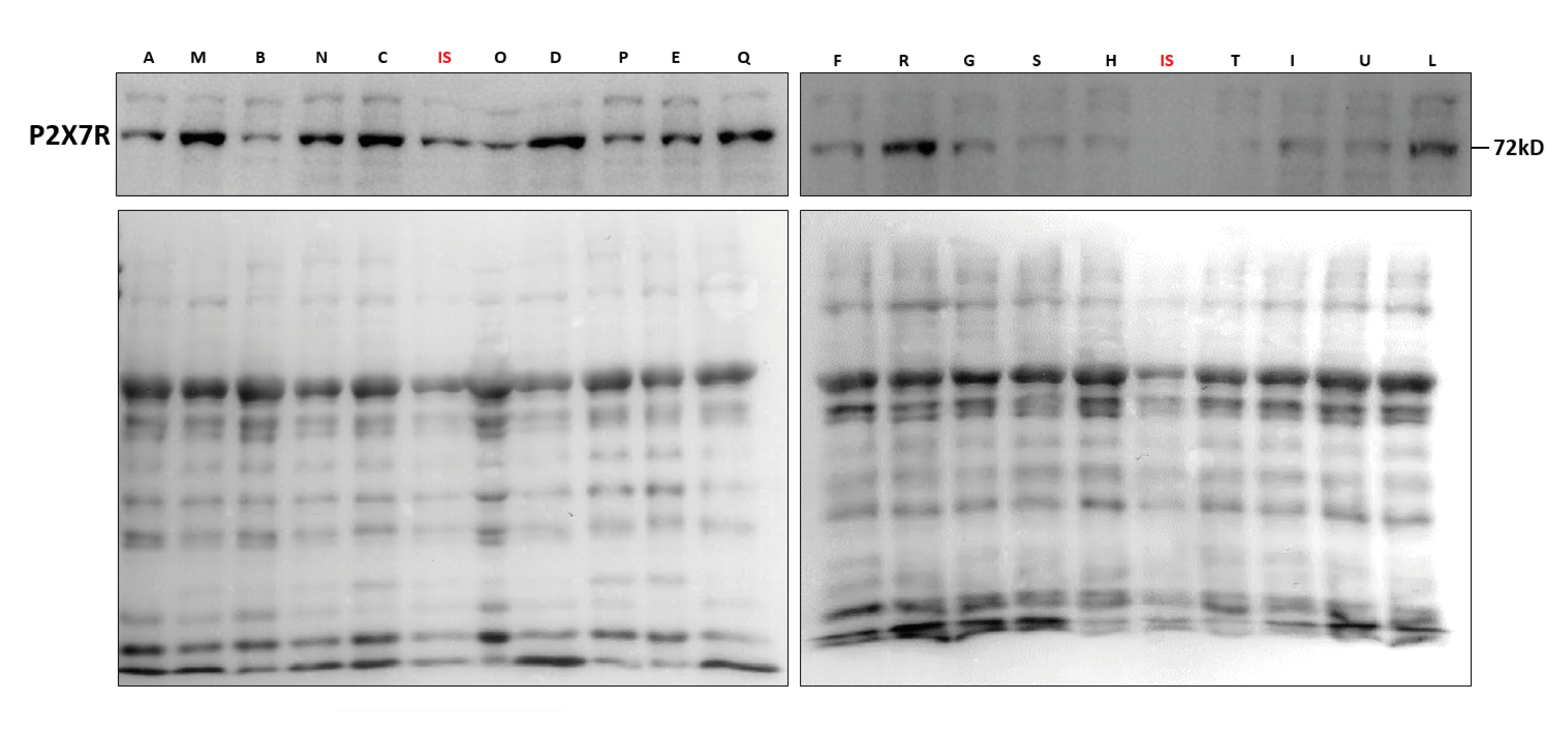

Supplement: Supplementary file 8 — Supplementary file8 (TIF 17812 KB) Supplementary Fig. 6 The P2XR7 levels are higher in muscle biopsies of slow progressing than fast progressing ALS patients. Representative Immunoblot images of P2XR7 in the Vastus Lateralis muscle lysates of fast and slow progressing ALS patients (see Supplementary Table 2). Immunoreactivity was normalised to the total amount of protein detected by the Stain-Free membrane activation system (BioRad). Given the analysis of two different membranes, the P2XR7 levels were analysed as follows: i) an internal standard (IS) representing the mix of all the samples in the experiment was loaded on each gel; ii) membranes were acquired at the same time; iii) the immunoreactivity of each sample was further normalised to the immunoreactivity of the IS. [file 18_2021_4070_MOESM8_ESM.tif]
