# Supplementary material for: Contingent intramuscular boosting of P2XR7 axis improves motor function in transgenic ALS mice
Source: Cell Mol Life Sci. 2021 Dec 22;79(1):7. doi: 10.1007/s00018-021-04070-8 (PMC8695421; doi:10.1007/s00018-021-04070-8)
Supplement: Supplementary file 7 — Supplementary file7 (DOCX 19 KB) [file 18_2021_4070_MOESM7_ESM.docx]

***Supplementary Table 2 List and features of the ALS patients whose muscle biopsies has been analysed***

| ID | Gender | Age onset | ^a^Diagnosis | Disease Onset | Date of Diagnosis | Type of onset | ^b^Site of the Onset | ^c^Site of Biopsies | Date of biopsy | ALSFR score at the diagnosis | Numerator  (*48- ALSFR score at diagnosis*) | Denominator (*months between onset and the diagnosis*) | ^d^ΔFRS (*Numerator/ Denominator*) |
| --- | --- | --- | --- | --- | --- | --- | --- | --- | --- | --- | --- | --- | --- |
| A | M | 62 | sALS | 01/2010 | 11/2015 | spinal | AAII | VLS | 03/2015 | 37 | 11 | 70 | 0,16 |
| B | M | 53 | sALS | 01/2014 | 02/2017 | spinal | AAII | VLS | 12/2016 | 42 | 6 | 37 | 0,16 |
| C | M | 56 | fALS | 04/2013 | 03/2014 | spinal | AAII | VLS | 07/2014 | 46 | 2 | 11 | 0,18 |
| D | M | 70 | sALS | 01/2013 | 09/2015 | spinal | AI_SX_ | VLS | 07/2015 | 43 | 5 | 28 | 0,18 |
| E | F | 50 | sALS | 02/2013 | 10/2015 | spinal | AAII | VLS | 10/2015 | 42 | 6 | 32 | 0,19 |
| F | M | 62 | sALS | 01/2014 | 02/2018 | spinal | AI_SX_ | VLS | 12/2017 | 38 | 10 | 49 | 0,20 |
| G | M | 63 | sALS | 04/2013 | 06/2014 | spinal | AI_sx_ | VLS | 04/2014 | 45 | 3 | 14 | 0,21 |
| H | M | 50 | sALS | 12/2012 | 01/2016 | spinal | AI_sx_ | VLS | 05/2015 | 36 | 12 | 49 | 0,24 |
| I | M | 40 | sALS | 09/2014 | 08/2015 | spinal | AI_dx_ | VLS | 08/2015 | 43 | 5 | 11 | 0,45 |
| L | F | 72 | sALS | 05/2017 | 05/2018 | spinal | AI_dx_ | VLS | 01/2018 | 40 | 8 | 12 | 0,67 |
| M | M | 61 | fALS | 02/2014 | 12/2014 | spinal | AI_sx_ | VLS | 10/2014 | 41 | 7 | 10 | 0,70 |
| N | F | 59 | sALS | 03/2014 | 01/2015 | spinal | AI_sx_ | VLS | 11/2014 | 41 | 7 | 10 | 0,70 |
| O | M | 55 | sALS | 10/2016 | 04/2017 | spinal | AAII | VLS | 07/2017 | 43 | 5 | 6 | 0,83 |
| P | F | 67 | sALS | 03/2017 | 11/2017 | spinal | AI_sx_ | VLS | 12/2017 | 41 | 7 | 8 | 0,88 |
| Q | M | 66 | sALS | 01/2012 | 11/2014 | spinal | AI_dx_ | VLS | 02/2015 | 17 | 31 | 34 | 0,91 |
| R | M | 50 | sALS | 07/2016 | 10/2017 | spinal | AI_sx_ | VLS | 07/2017 | 34 | 14 | 15 | 0,93 |
| S | M | 55 | sALS | 01/2018 | 10/2018 | spinal | AAII | VLS | 09/2018 | 38 | 10 | 9 | 1,11 |
| T | M | 57 | sALS | 01/2014 | 05/2015 | spinal | AAII | VLS | 05/2015 | 27 | 21 | 16 | 1,31 |
| U | M | 60 | sALS | 09/2017 | 12/2017 | spinal | AAII | VLS | 06/2017 | 44 | 4 | 3 | 1,33 |

***^a^****sALS = sporadic ALS, fALS = familial ALS;* ***^b^***AAII = *Lower Limbs*, AI_SX_ = *Left Lower Limb;* AI_dx_ = *Right Lower Limb;* ***^c^****VLS = Vastus Lateralis Sinistro (left).* ***^d^****The higher is the* ΔFRS, the faster is the disease progression.
